# A Useful Guide to Lectin Binding: Machine-Learning Directed Annotation of 57 Unique Lectin Specificities

**DOI:** 10.1021/acschembio.1c00689

**Published:** 2022-01-27

**Authors:** Daniel Bojar, Lawrence Meche, Guanmin Meng, William Eng, David F. Smith, Richard D. Cummings, Lara K. Mahal

**Affiliations:** † Department of Chemistry and Molecular Biology and Wallenberg Centre for Molecular and Translational Medicine, University of Gothenburg, Gothenburg, Sweden 405 30; ‡ Biomedical Chemistry Institute, Department of Chemistry, New York University, 100 Washington Square East, Room 1001, New York, New York 10003, United States; § Department of Chemistry, University of Alberta, Edmonton, Canada, T6G 2G2; ∥ Department of Biochemistry, Glycomics Center, School of Medicine, Emory University, Atlanta, Georgia 30322, United States; ⊥ Department of Surgery, Beth Israel Deaconess Medical Center, Harvard Medical School, Boston, Massachusetts 02115, United States

## Abstract

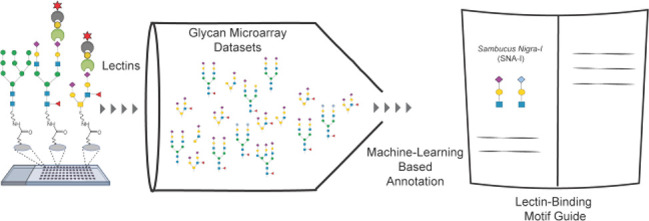

Glycans are critical to every facet of biology and medicine, from viral infections to embryogenesis. Tools to study glycans are rapidly evolving; however, the majority of our knowledge is deeply dependent on binding by glycan binding proteins (e.g., lectins). The specificities of lectins, which are often naturally isolated proteins, have not been well-defined, making it difficult to leverage their full potential for glycan analysis. Herein, we use a combination of machine learning algorithms and expert annotation to define lectin specificity for this important probe set. Our analysis uses comprehensive glycan microarray analysis of commercially available lectins we obtained using version 5.0 of the Consortium for Functional Glycomics glycan microarray (CFGv5). This data set was made public in 2011. We report the creation of this data set and its use in large-scale evaluation of lectin–glycan binding behaviors. Our motif analysis was performed by integrating 68 manually defined glycan features with systematic probing of computational rules for significant binding motifs using mono- and disaccharides and linkages. Combining machine learning with manual annotation, we create a detailed interpretation of glycan-binding specificity for 57 unique lectins, categorized by their major binding motifs: mannose, complex-type *N*-glycan, *O*-glycan, fucose, sialic acid and sulfate, GlcNAc and chitin, Gal and LacNAc, and GalNAc. Our work provides fresh insights into the complex binding features of commercially available lectins in current use, providing a critical guide to these important reagents.

## Introduction

Carbohydrates (i.e., glycans) are involved in every facet of life from the cell walls of bacteria to the signals that start inflammatory cascades in humans.^1^ While our understanding of other biomolecules, such as DNA and RNA, has expanded exponentially due to the advent of new analytical technologies, glycans have remained understudied due to the lack of convenient analytical tools. Nature solved the problem of glycan identification by designing noncatalytic glycan binding proteins called lectins, which recognize well-defined epitopes within a glycan. Lectins, which can be found in all organisms, are often isolated for commercial use from plants and have long been used as tools for analysis of the mammalian glycome.^2^ The connection between the biological functions of plant lectins and their binding of mammalian epitopes is not well understood. However, these probes have commonly been used to probe glycosylation. Our earliest understanding of blood group antigens comes from agglutination studies using lectins to determine blood type.^3^ More modern methods that leverage lectins as analytical tools include lectin histology and enzyme-linked lectin assays (ELLA).^3^ Lectin microarrays, in which a panel of lectins (10s to >100) and other carbohydrate-binding probes (e.g., antibodies) are printed on a solid support, are now routinely used in glycomics.^4−6^ This high-throughput method has identified glycans involved in a variety of systems from melanoma metastasis to viral host response.^7−9^

Although lectins are useful tools, they have suffered from a lack of detailed definition of their binding requirements, which hampers their analytical utility. Traditional methods of defining lectin specificity have involved inhibition assays with monosaccharides, binding assays with a limited set of potential ligands, or in a few cases, crystal structures with disaccharide or monosaccharide binders. These are often the data commercial suppliers of lectins provide about binding specificities, typically pointing to mono- or disaccharide motifs. In more recent years, glycan microarrays, in which the binding of probes to hundreds of glycans are interrogated simultaneously, have been used to identify more detailed glycan-binding specificities.^10−12^ In 2011, the Mahal Laboratory, in collaboration with the Consortium for Functional Glycomics (CFG), collected glycan microarray data for 116 commercially available lectin preparations using version 5.0 of the Consortium for Functional Glycomics glycan microarray (CFGv5). This data set was made public via the CFG database in 2011, and subsets of this data have been used to perform cross-platform comparisons of glycan microarrays,^13,14^ model lectin-glycan interactions,^15^ and create new bioinformatic methods for glycan microarray analysis.^16−19^ Herein, we analyze this data set, using a combination of machine learning and expert manual annotation, to provide a useful guide to the glycan-binding specificities of these commercial probes.^13^

Connecting binding events with glycan substructures or motifs has attracted considerable interest for the purpose of identifying glycan-receptor interactions. Related work has focused on analyzing lectins and their cognate binding motifs from a protein sequence or structural perspective.^20,21^ Under the assumption that similar sequences and/or structures bind similar ligands, lectins can be grouped into classes with potentially shared binding properties. This can aid lectin categorization, annotation, and utility for researchers working with these lectins. This concept has recently been taken further with LectinOracle,^22^ a deep learning algorithm that utilizes protein and glycan sequences to predict lectin specificity. To annotate the specificities of glycan binding proteins more directly from glycan microarray data, multiple algorithms including frequent subtree mining^18,23^ and motif based approaches have been developed.^16,17^ Yet the high diversity and nonlinearity of glycans has stymied the large-scale evaluation of subtle, interpretable binding motifs in glycan array data to date. To overcome this, we have leveraged the recent introduction of machine learning into glycobiology.^24^ By mapping inputs (glycan sequences) to outputs (lectin-glycan binding), machine learning algorithms can ascertain the most important features (i.e., sequence motifs) that predict lectin–glycan binding. Importantly, this is performed on a scale that is vastly larger than manual annotation and also enables the analysis of highly complex feature combinations to obtain insights into more subtle influences of the co-occurrence of glycan motifs.

We further engaged in feature engineering by combining hand-crafted features that are domain-relevant (e.g., the presence of Lewis A or terminal sialosides) with systematic probing for all observed sequence motifs of various lengths. This procedure improves the interpretability of the resulting machine learning models, which have traditionally been labeled as uninterpretable “black boxes”. We then used these features, our machine learning models, and iterative manual annotation to establish logical, interpretable rules for each lectin that best explained lectin–glycan binding behavior to further facilitate “white box” machine learning and extract lectin binding specificities. Upon removal of duplicate lectins (e.g., preparations of the same lectin from different sources) and lectins with poor binding, our analysis provides detailed annotation of 57 unique commercially available lectins. While lectin binding specificities are routinely analyzed on the basis of glycan array data, the most common mode of analysis is to explain what is being bound, for instance, via shared or enriched motifs. Here, we expand on this concept by also considering glycans that are not bound by a lectin, both in our machine learning analysis and in the subsequent expert annotation. This procedure allowed us to identify additional binding determinants that, for instance, inhibit binding to a preferred motif when present and overall improve the precision and usefulness of our lectin binding annotation. The results of our work give a clear view into the binding profiles of these carbohydrate-binding reagents and demonstrate that they are selective in their epitope binding. Overall, our work provides a useful guide to anyone analyzing glycans using this reagent class and sets the stage for more advanced interpretation of studies using lectins.

## Results and Discussion

### Study Design

In 2011, our laboratories analyzed the glycan binding specificities of commercially available lectins from various sources (116 total) using the CFG glycan microarray, version 5.0. (CFGv5). Although this data set was made publicly available soon thereafter, and parts of this data set were analyzed by multiple laboratories,^13,16−19^ details on the creation of this data set were never reported. The CFGv5 array contained 611 glycan structures, including those representing both *N-* and *O-*glycans (Table S1). Of the epitopes on CFGv5, 22.3% represented *N-*linked glycans, with the exception of hybrid *N*-linked sugars, which were not present on this array. Another 18.5% of structures represented *O*-linked glycans. This subset focused on short *O-*glycans of various core structures, with little representation of more elaborate structures. The remaining array epitopes were either terminal or fragmented glycans, which can appear on either *N-* or *O-*linked carbohydrates, or select glycolipid structures (59%). All glycans were attached to the slides via NHS-coupling to either aliphatic amine linkers of varying lengths or the amino termini of amino acids (serine, threonine, asparagine, glycine).^4,10^ Each printing of the glycan microarrays was accompanied by a quality control assessment, details of which can be found in a review by Heimburg-Molinaro et al.^25^

**Figure 1:**
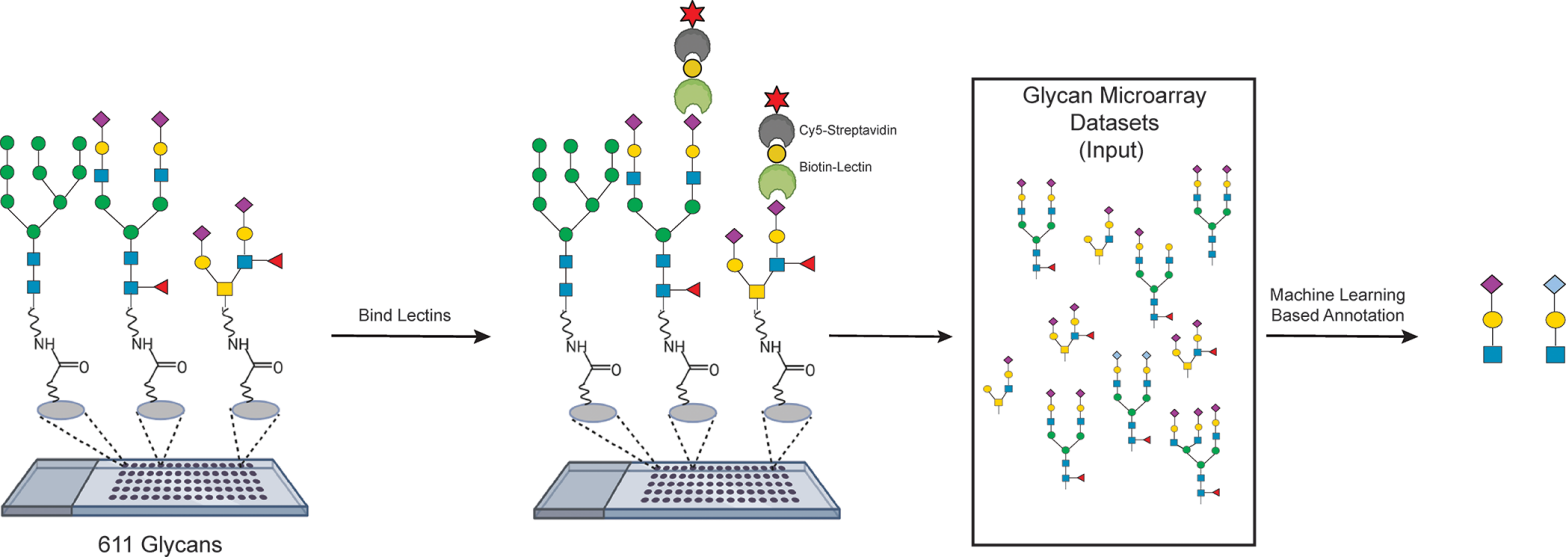
Glycan microarray data sets generated with the Consortium for Functional Glycomics glycan microarray version 5 (CFGv5) containing 611 unique glycans. Biotinylated lectins (116 total) were incubated with the arrays at varying concentrations, followed by incubation with Cy5-Streptavidin. Slides were processed and scanned, and data were extracted. For each array, the average fluorescence for each glycan (*n* = 4 spots) was obtained. Array data sets were then used as input for machine learning and *Z*-score analysis to identify motifs.

We analyzed 116 commercially available lectin preparations, including multiple lots of the same lectin from a variety of sources (Table S2). Where available, the biotinylated versions of lectins were purchased. Lectins that were available only in an unmodified form were biotinylated for analysis using standard protocols (AOL, CF, PA-IL, PAA, SVAM, TJA-I, TJA-II). In general, lectins were incubated with the array at 3–6 concentrations ranging from 0.1 to 100 μg/mL, and binding was detected via Cy5-labeled streptavidin (Figure 1).^13^ Analyzing several concentrations of a lectin allows for better separation between the strong and weak binders through sampling of a greater cross-section of the binding interactions. For each array, fluorescence data were extracted for the glycans, which were represented by six spots per glycan. The average fluorescence of four spots, excluding the high and low values, was obtained for each glycan. Glycans that did not show significant variance across lectins were removed from the analysis (for an annotated list of glycans see Table S1). Of the 116 lectin preparations, 42 were duplications of a lectin from a different source (e.g., WGA from Sigma, WGA from Vector Laboratories). An additional 15 lectins displayed low binding activity and were thus excluded from our motif analysis (Table S2). The *Anguilla anguilla* agglutinin (AAA), from eels,^26^ and *Vicia graminea* lectin (VGA)^27^ both showed binding that strongly disagreed with the literature. AAA, which is known to bind terminal Fucα1,2-containing glycans, bound only chitin and related structures on the array. VGA which binds the N-antigen and clustered Galβ1–3GalNAc antigens^2^ was found in our analysis to bind high mannose glycans. No binding to Galβ1–3GalNAc glycans on the array was observed. These results may be due to presentation issues, discussed in more detail in the conclusions.^13,14,28^ Due to the incongruous binding of these lectins, they were removed from our analysis. After exclusions, we annotated the binding specificities of 57 unique lectins.

Binding motifs were annotated using a combination of two different approaches. First, we applied machine learning to identify the predominant binding motif(s) for each lectin. As input features for machine learning analysis, we curated a collection of 68 motifs (e.g., blood group B, terminal sialosides, etc.) seen in the CFGv5 (Table S1) and used these in tandem with all observed mono- and disaccharide motifs, including all observed linkages. These were used to generate binding rules and associated *p* values (Tables S3 and S4) that best explained the experimental results. In all of our analyses, the rules observed with machine learning were considered the predominant binding rules.

The machine learning analysis only gives a part of the picture, as only previously specified features can be detected as relevant. To complement our machine learning approach, we generated a combined *Z*-score analysis of the glycan microarray data and used that for further manual annotation (Table S5). *Z* scores measure the deviation of individual glycans as binders (as reflected in fluorescence) from the mean. Using this metric as a measurement of binding assumes that the majority of glycans on the array are not bound by the lectin, and thus the mean fluorescence indicates no true binding. We used Stouffer’s Z-score method to combine data sets of multiple concentrations for each lectin tested. This gave a single metric (*Z*_s_) for lectin binding to each glycan.^29^ We set *Z*_s_ = 1.645 as our threshold for binding as this corresponds to a one-tailed *p* value = 0.05, i.e., the 95% confidence interval.^30^ In evaluating a binding motif, we first applied the machine learning rules. We then examined glycans following the rules that either bound or did not bind (based on *Z*_s_ score) and looked for features that could account for the difference. We used this information to annotate the predominant binding specificity. We next looked at glycans that did not follow the machine learning rules but were nonetheless bound based on *Z*-score analysis. We again looked for features that could account for binding, or lack thereof, and annotated these as additional binding motifs. Combining machine learning with manual annotation gave a more complete description of binding than either method alone.

### Overview of Glycan Binding Profiles

**Figure 2:**
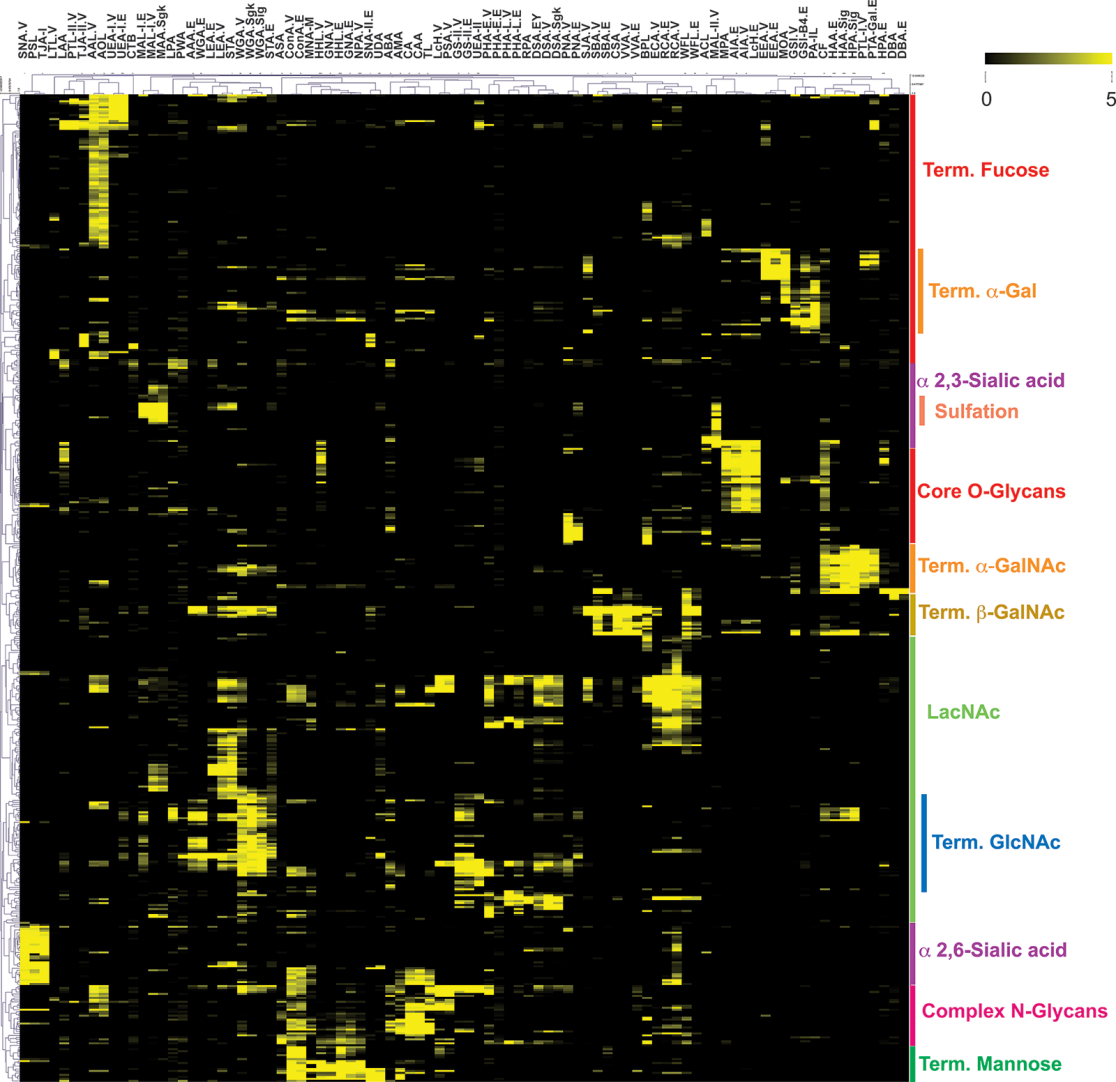
Heatmap of glycan binding data (*Z*_s_ scores). Data were clustered using the Pearson correlation coefficient and average linkage analysis. Yellow indicates *Z*_s_ > 1.645 (e.g., 95% confidence interval for binding); black indicates no significant binding. Rough annotations of glycan motifs are shown on the right. Term. = terminal.

To analyze the potential overlap in glycan binding motifs of the commercially available lectins, we hierarchically clustered our *Z*_s_ data sets via average linkage analysis using the Pearson correlation coefficient as our distance metric. The heatmap of the cluster is shown in Figure 2. From this heatmap, we can clearly observe that lectins bind distinct subsets of the glycome. Generally speaking, lectins clustered according to their major binding motifs.

**Figure 3:**
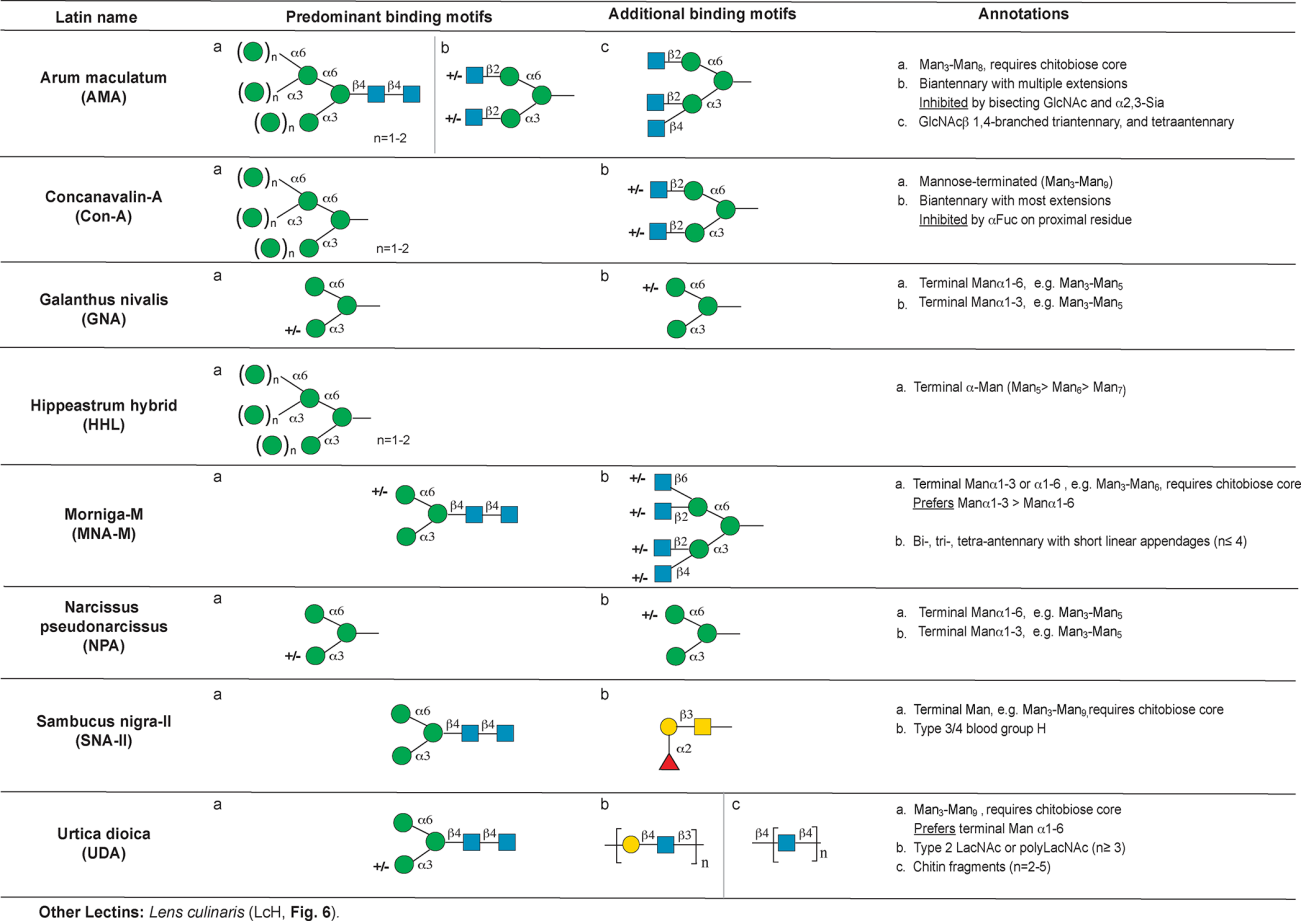
Annotation of predominant mannose binding lectins. Other lectins that also bind mannose are listed at the bottom of the figure. Abbreviations and symbols: Mannose (Man, green circles), sialic acid (Sia, pink diamonds), *N-*acetylglucosamine (GlcNAc, blue squares), *N-*acetyllactosamine (LacNAc), fucose (Fuc, red triangles), galactose (Gal, yellow circles), *N-*acetylgalactosamine (GalNAc, yellow squares). The Symbolic Nomenclature for Glycans (SNFG) is used.

**Figure 4:**
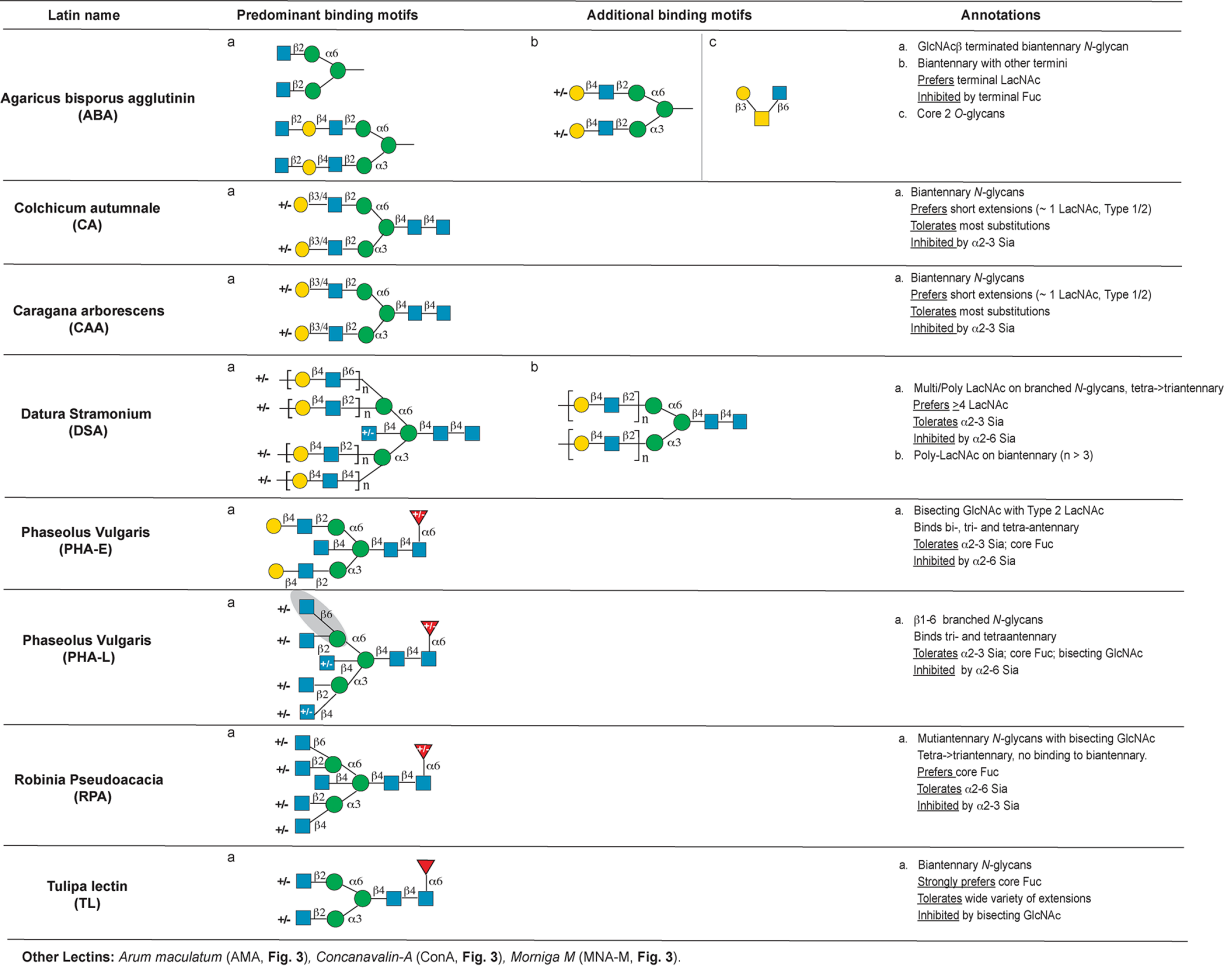
Annotation of lectins binding complex *N*-glycan motifs.

**Figure 5:**
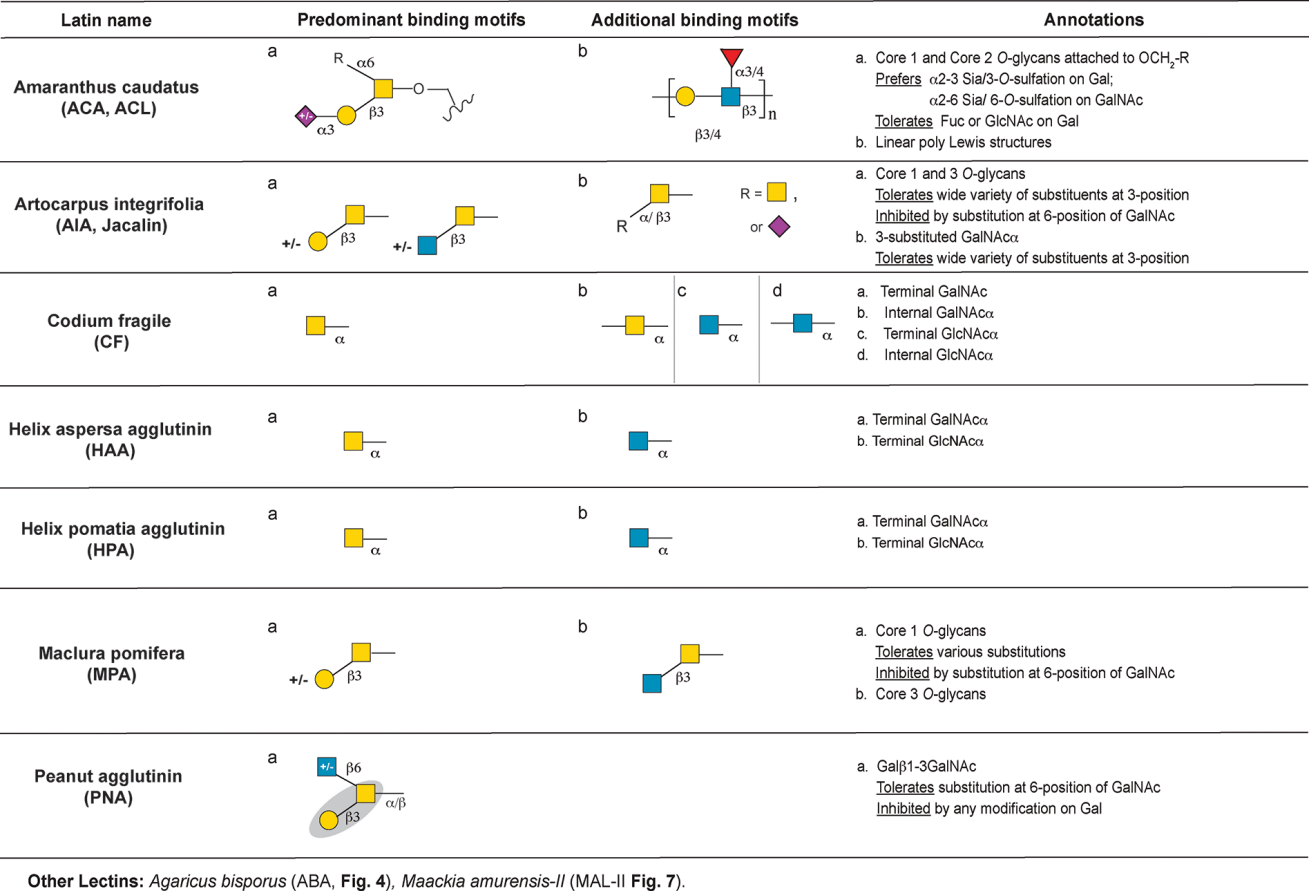
Annotation of lectins binding core *O*-glycan motifs.

**Figure 6:**
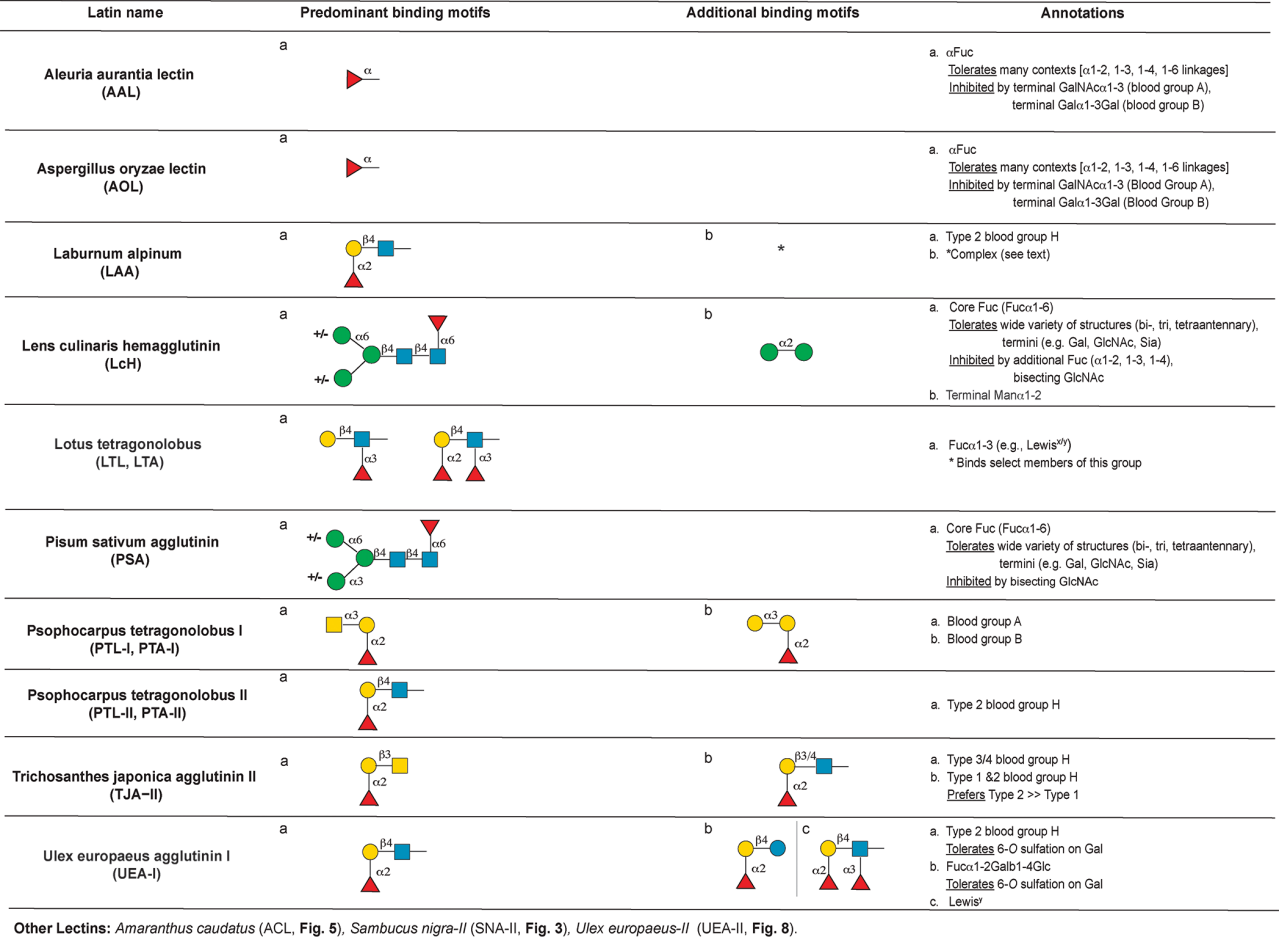
Annotation of fucose-binding lectins.

**Figure 7:**
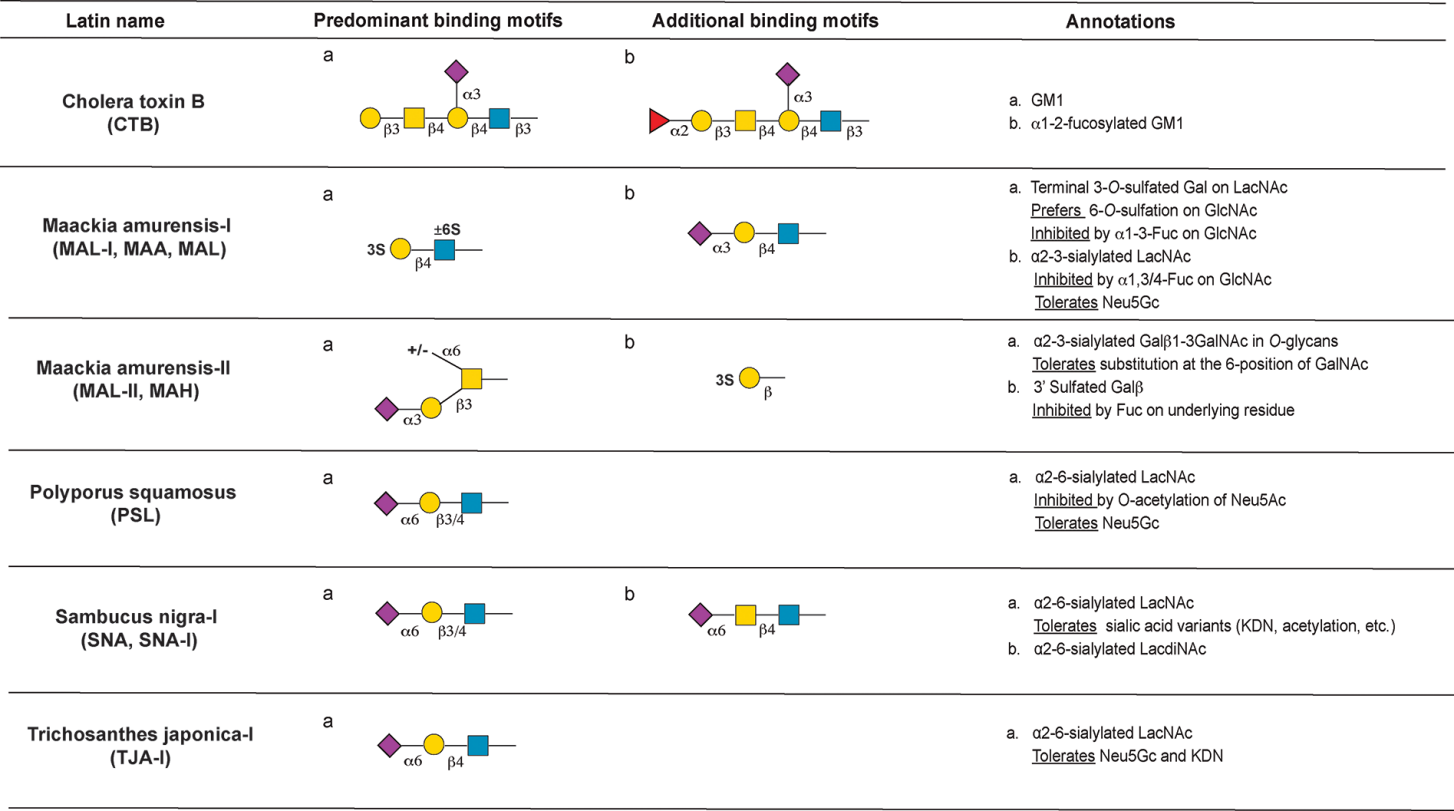
Annotation of sialic acid and sulfate binding lectins.

**Figure 8:**
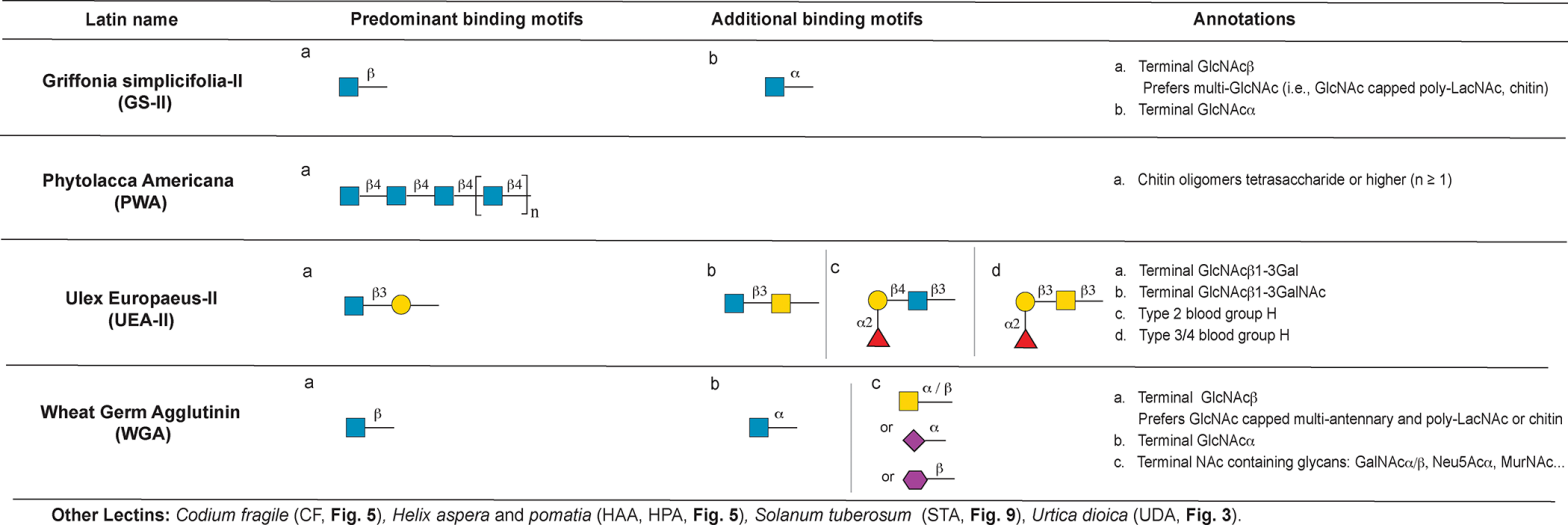
Annotation of GlcNAc and chitin binding lectins.

**Figure 9:**
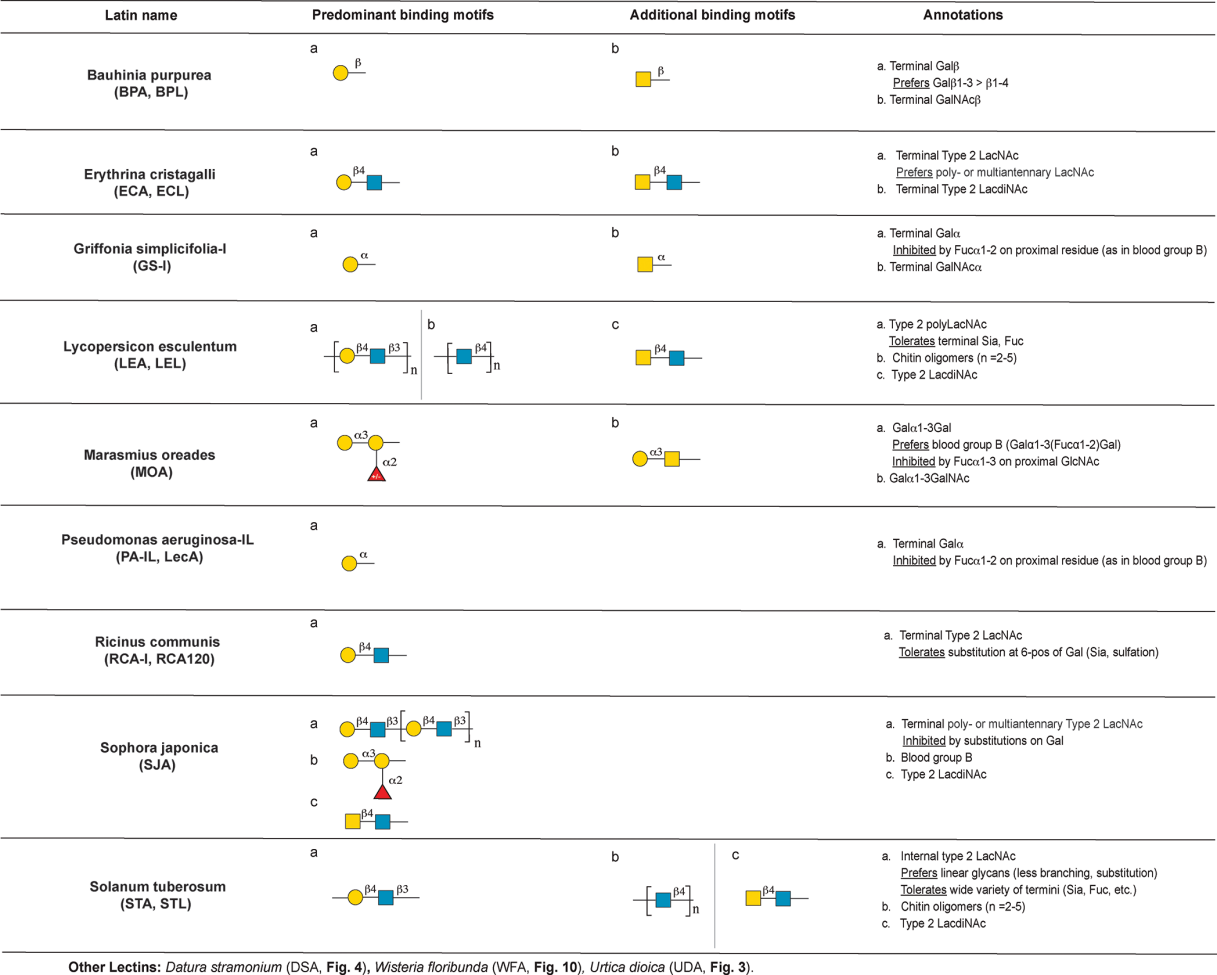
Annotation of Gal and LacNAc binding lectins.

**Figure 10:**
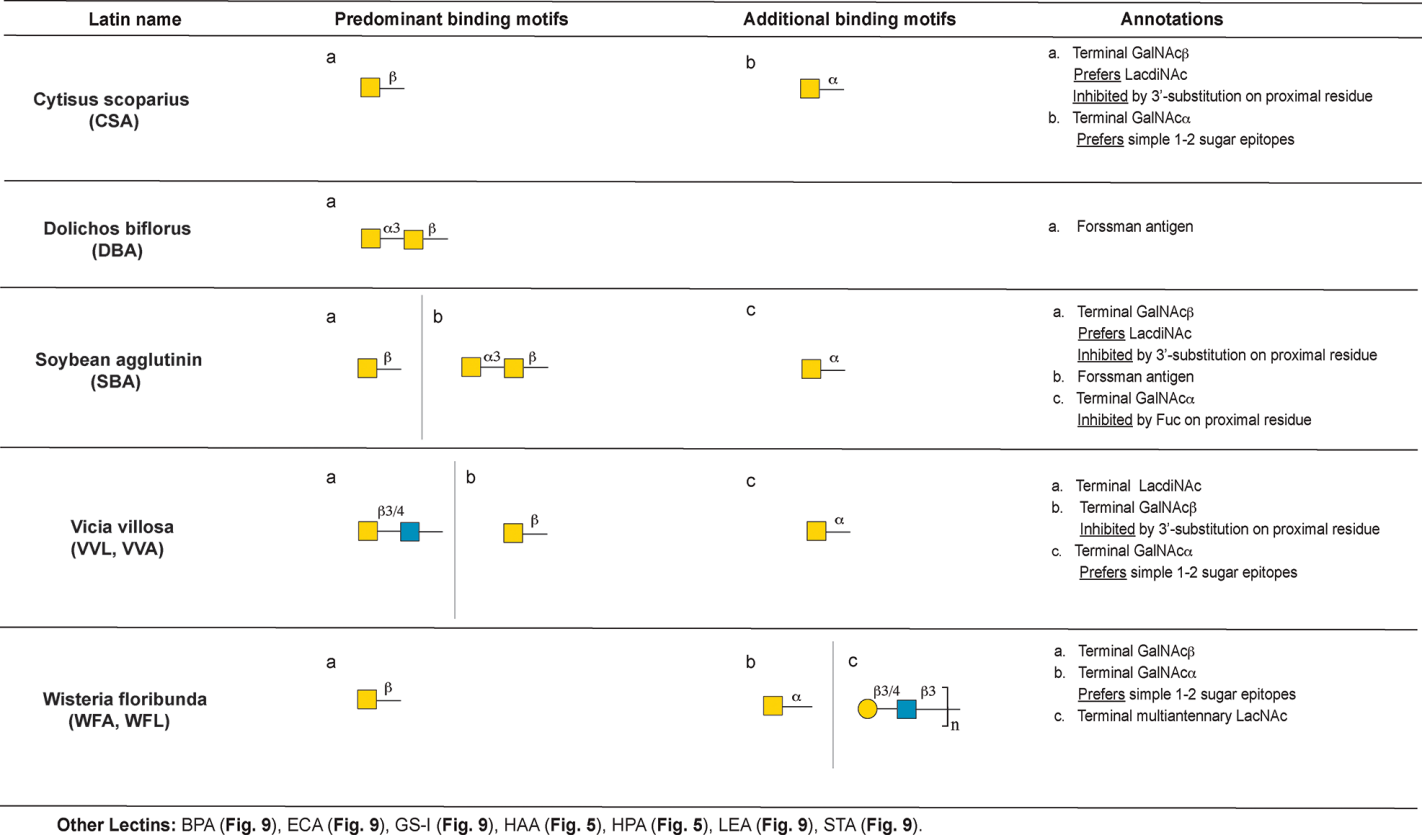
Annotation of GalNAc binding lectins.

In the following sections, we provide a detailed annotation of the glycan binding motifs of commercially available lectins revealed by our glycan microarray analysis. In most cases, although the rough specificities of these lectins are known, our analysis has revealed more subtle binding preferences. However, in some instances we have identified dramatic differences between the rough specificities identified by earlier techniques and what is observed through our analysis. We have organized the lectins by commonly referenced glycosylation motifs to make our analysis more useful to the scientific community. For each motif, we provide a table of lectins with the predominant and additional binding motifs outlined using the Symbolic Nomenclature for Glycans (SNFG).^31^ Lectins are organized by their predominant motifs; however each table also lists other lectins that can bind that motif. In the tables, features that enhance binding (denoted by *Prefers*), those tolerated by the lectin but which have no or little impact on binding (denoted by *Tolerates*), and features that inhibit binding (denoted by *Inhibited by*) are indicated. We organized lectins using the following motifs: mannose (Figure 3), complex *N*-glycan (Figure 4), core *O*-glycans (Figure 5), fucose (Figure 6), sialic acid and sulfate (Figure 7), terminal *N-*acetylglucosamine (GlcNAc) and chitin (Figure 8), terminal galactose (Gal) and *N-*acetyllactosamine (LacNAc, Figure 9), and terminal *N-*acetylgalactosamine (GalNAc, Figure 10). In general, lectins are usually referred to by the Latin name of the plant from which they are derived, followed by the word agglutinin or lectin. This is typically shortened to a three to four letter acronym (e.g., wheat germ agglutinin = WGA). Where a lectin is commonly referred to as either the agglutinin or lectin, both acronyms are given. Detailed discussion of the binding specificities of the lectins can be found in the text below.

### Mannose Binding Lectins

High mannose epitopes are among the least processed *N-*glycans, resulting from trimming of the Glc_3_Man_9_GlcNAc_2_-structure that is transferred cotranslationally by oligosaccharyltransferase. Man_7_-Man_9_, i.e., high mannose, all contain terminal α1,2-mannose residues (Figure S1A). Further trimming of these structures results in oligomannose structures Man_5_–Man_6,_ characterized by exposure of the trimannosyl core [Manα1–6(Manα1–3)Man]. This trimannosyl core is also exposed in hybrid *N-*glycan structures, although these are not represented on CFGv5. Mannose is also found as a direct modification of serines and threonines in noncanonical *O*-linked glycans (*O*-mannosylation).^32^ The following lectins predominantly recognized mannose-based epitopes on the array: *Arum maculatum* agglutinin (AMA), *Concanavalin-A* (ConA), *Galanthus nivalis* lectin (GNA, GNL), *Hippeastrum hybrid* lectin (HHA, HHL), *Morniga M* agglutinin (MNA-M), *Narcissus pseudonarcissus* lectin (NPA), *Sambucus nigra* agglutinin II (SNA-II), and *Urtica dioica* lectin (UDA, Figure 3). In addition, *Lens culinaris* hemagglutinin (LcH, Figure 6) also binds this motif. A more detailed analysis of these lectins is given below.

#### *Arum maculatum* (AMA)

The lectin from *Arum maculatum* (AMA) is noted to bind both *N-*acetyllactosamine (LacNAc) and mannose.^33,34^ We observe two predominant glycan binding motifs: mannose-terminated *N-*glycans and biantennary structures (Figure 3). Perhaps due to the complexity of the two motifs, no binding rules were identified by machine learning, but these motifs were clear in manual annotation. For the mannose-terminated glycans, AMA recognizes Man_3_–Man_8_. In contrast to data obtained using mannose fragments in inhibition studies,^35^ recognition requires the chitobiose core (GlcNAcb1–4GlcNAc). An exposed α1,3- or α1,6-mannosyl residue is observed in all binders, thus Man_9_ is not recognized. Biantennary glycans were also among the top binders. AMA can recognize triantennary *N-*glycans with β1,4-branching, and some tetraantennary with lower affinity. Although AMA tolerates a wide range of terminal structures (α2,6-sialic acid, fucose, Gal, GlcNAc, etc.) on bi-, β1,4-tri-, and tetra-antennary, its binding is inhibited in the presence of bisecting GlcNAc (previously observed in ref (35)), or α2,3-sialic acid (not previously known). Previous work identified core fucose as an enhancer of binding, but this was not observed when comparing closely related ligands on our arrays.^35^

#### Concanavalin-A (ConA)

Isolated in 1936, ConA is perhaps the most commonly used lectin.^36^ ConA is often annotated as a high-mannose binding lectin,^37^ although its true specificity is more complex. ConA from two sources was evaluated and had very similar bindings, showing terminal α-mannose as the predominant binding motif (Figure 3). ConA recognizes Man_3_–Man_9_, and core chitobiose is not necessary for recognition. Consistent with previous work, ConA also recognized a wide range of biantennary *N-*glycans, tolerating multiple extensions (sialylated LacNAc, fucose, Gal, GlcNAc, etc.).^13^ However, our analysis revealed that binding is inhibited by α1,2- or α1,3-linked fucose attached to the proximal glycan at the termini (e.g., as in Galβ1,4 (Fuc α1,3) GlcNAc).

#### *Galanthus nivalis* Lectin (GNA, GNL) and *Narcissus pseudonarcissus* Lectin (NPA, NPL)

GNA and NPA are both isolated from bulbs (snowdrop^38^ and daffodil,^39^ respectively). On the CFGv5 array, the binding specificities of these two lectins overlap very closely and show no clear differences in binding determinants. The literature reports that GNA prefers terminal α1,3-linked,^40^ whereas NPA prefers α1,6-linked mannose.^41^ However, machine learning identifies terminal Manα1–6 as the top motif for recognition by both lectins (Figure 3). Terminal Manα1–3 is also recognized; however, it exhibits weaker binding. Man_3_–Man_5_ are preferred over Man_6_–Man_8_, and little to no binding is observed to terminal α1,2-linked mannose, in contrast to studies using multimerized α1,2-epitopes.^42^ Our analysis identifies short LacNAc terminated N-glycan epitopes as an additional binding determinant for NPA and GNA. GNA from two different sources showed highly similar binding patterns.

#### *Hippeastrum hybrid* Lectin (HHA, HHL)

*Hippeastrum hybrid* lectin (HHA, HHL) is known to bind terminal mannose.^39^ Consistent with this, HHL from two sources (EY and Vector) was seen to predominantly bind terminal α-mannose (Figure 3). This lectin shows the highest affinity for the mannose core trisaccharide (Manα1,6(Manα1,3)Man) and does not require the chitobiose core. Man_5_–Man_8_ are bound, with Man_5_ < Man_6_ < Man_7_ < Man_8_.

#### Morniga-M (MNA-M)

One of two lectins isolated from the bark of the black mulberry tree (*Morus nigra*), Morniga M (MNA-M), was identified as a mannose specific lectin. Further characterization by frontal affinity chromatography found it to prefer Man_3_–Man_6_, with unsubstituted Manα1,3Man in the best binders.^43^ Our analysis of this lectin is in keeping with this earlier work. The best binders for MNA-M all contain an unsubstituted Manα1–3 or Manα1–6, although machine learning shows a requirement for the chitobiose core (Figure 3). We also observed additional binding to bi-, tri-, and tetra-antennary *N-*glycans with short linear appendages (*n* = 4 or less).

#### *Sambucus nigra* Agglutinin II (SNA-II)

SNA-II is one of several lectins isolated from elderberry bark.^44^ SNA-II is reported to be a terminal GalNAc/Gal binder;^45^ however these studies used only a small number of simple sugar structures (mono- and disaccharides) to probe the binding motif. Our machine learning analysis indicates that the top motif for SNA-II is terminal mannose (Man_3_–Man_9_) containing the chitobiose core as an essential component (Figure 3). This lectin also binds type 3 and 4 blood group H antigens (e.g., Fucα1,2Galβ1,3GalNAc). In lectin microarray analysis, this lectin often clusters with TJA-II, which also binds type 3 and 4 blood group H.^46^ Binding is also observed to some terminal type 2 LacdiNAc glycans (GalNAcb1,4GlcNAc).

#### *Urtica dioica* (UDA)

UDA is annotated as a GlcNAc binding lectin;^47^ however our analysis reveals that its predominant binding is terminal Manα1,6 (Figure 3). UDA will recognize Man_3_–Man_9_ and requires the chitobiose core. Chitin fragments (i.e., (−GlcNAcβ1,4GlcNAc−)_*n*_) and polymers of type 2 LacNAc (*n* ≥ 3 disaccharides) are also bound, but at a lower apparent affinity. Our analysis fits modeling of these ligands into the UDA active site.^15^

### Complex N-Glycan Binding Lectins

In *N-*glycan processing, high mannose *N-*glycans are trimmed to Man_5_, and a β1,2-GlcNAc is appended to the exposed core α1,3-mannose to form hybrid structures (Figure S1). The trimannosyl epitope on the core α1,6-mannose is subsequently removed, and the core α1,6-mannose branch is further elaborated to give biantennary *N-*glycans. ConA and AMA recognize biantennary *N-*glycans; however, as it is not their predominant binding motif, they are discussed with the high mannose lectins (Figure 3). Lectins from *Agaricus bisporus* (ABA, ABL), *Colchicum autumnale* (CA), *Caragana arborescens* (CAA), and *Tulipa* lectin (TL) predominantly recognized biantennary *N-*glycans epitopes on the array (Figure 4)

Biantennary structures can be further elaborated to tri- and tetra-antennary structures. In triantennary glycans, branching can occur at either the β1,4 position (GlcNAcβ1,4Manα1,3Man) or the β1,6 position (GlcNAcβ1,6Manα1,6Man) on the trimannosyl core.^48^ Additionally, a GlcNAc can be added β1,4 to the central mannose of the core, resulting in the bisecting GlcNAc motif, and fucosylation of the chitobiose core is common (Figure S1). Several lectins are specific for these complex epitopes including *Datura stramonium* (DSA), *Phaseolus vulgaris-*E (PHA-E), *Phaseolus vulgaris*-L (PHA-L), and *Robinia Pseudoacacia* (RPA). In addition, Morniga M can also bind select complex epitopes (Figure 3). Core fucose binding lectins are included in Figure 6. A more detailed analysis of the lectins binding complex *N-*glycan epitopes is given below (Figure 4).

#### *Agaricus bisporus* Agglutinin (ABA, ABL)

Although the agglutinin from *Agaricus bisporus* (ABA, ABL) is thought to bind predominantly to *O*-glycans,^49^ ABA has been shown to display dual specificity, binding both agalactosylated biantennary and *O*-glycans.^50^ Our machine learning analysis identifies agalactosylated (GlcNAcβ-terminated) biantennary *N*-glycans as its predominant binding motif (Figure 4). ABA also recognizes biantennary *N*-glycans with other termini, especially terminal LacNAc; however, our analysis shows that this binding is inhibited by fucosylation on or near the termini. We also observed some *O*-glycan binding. In contrast to the literature indicating that ABA binds to core 1,^50^ our analysis identifies core 2 as the preferred motif, and little binding to core 1 epitopes is observed.

#### *Colchicum autumnale* (CA) and *Caragana arborescens* (CAA)

CA, from meadow saffron, and CAA, the major lectin from the pea tree *Caragana arborescens*, are both annotated as GalNAc binders, and little is known beyond this rough specificity determination.^51−53^ These two lectins show almost identical binding patterns by machine learning (Figure 4). Both bind biantennary *N*-glycans with short extensions (i.e., ∼ 1 of either type 1 [Galβ1,3GlcNAc] or type 2 [Galβ1,4GlcNAc] LacNAc). CA and CAA tolerate most substitutions (e.g., fucose or α2,6-sialic acid), but binding is inhibited by α2,3-sialic acid. Although terminal blood group B is bound, blood group A, which contains a terminal GalNAc, is not recognized. Although the Forssman pentasaccharide has been used as an inhibitory sugar,^53^ we observe no recognition of GalNAc or Forssman type epitopes on the array, arguing that these lectins are not predominantly GalNAc binders

#### *Datura stramonium* (DSA)

Isolated from Jimson weed (*Datura stramonium*), DSA was initially identified as a chitin-binding lectin,^54^ although further characterization revealed preferential binding to type 2 polylactosamine (polyLacNAc, [Galβ1,4GlcNAc]_*n*_) and β1,6-branched *N-*glycans.^55,56^ Our analysis is largely consistent with these findings, identifying branching structures with four or more type 2 LacNAc repeats in total as the predominant binding motif. These can be either on different multiantennary branches, or polyLacNAc chains (*n* ≥ 3 repeats) on biantennary *N-*glycans (Figure 4). Tetra-antennary *N-*glycans containing type 2 LacNAc are preferred to triantennary with similar epitopes, indicating that the binding affinity for DSA increases with higher branching. In contrast to the literature, we observe no preference for β1,6- over β1,4-triantennary structures. This also contradicts the analysis of this data set by a motif mining algorithm.^17^ Machine learning also indicates a preference for β1,6 structures (Table S4). However, direct comparison of the glycans on this array that are precisely matched except for the branching (461/462, 465/466, Table S5) shows a clear preference for β1,4-triantennary structures when all other factors are equal and reveals the advantage of our mixed machine learning and expert annotation. Biantennary *N-*glycans with long polyLacNAc chains (*n* ≥ 3 LacNAc residues) are weaker binders. Bisecting GlcNAc and terminal α2,3-sialic acids are tolerated, but α2,6 sialic acid inhibits lectin binding. DSA from three different sources (Vector, EY, Seikagaku) showed consistent binding patterns. As the CFG v5.0 array contains no representations of type 1 LacNAc of a similar length or presentation, we cannot assess whether DSA binds type 1 polyLacNAc epitopes.

#### *Phaseolus vulgaris*-Erythroagglutinating (PHA-E) and *Phaseolus vulgaris*-Leukoagglutinating (PHA-L)

*Phaseolus vulgaris*, also known as the kidney bean, has at least five isolectins.^2,57,58^ The two main isolectins of *Phaseolus vulgaris*, PHA-E and PHA-L, bind complex *N-*glycan epitopes with PHA-E annotated as a bisecting GlcNAc specific lectin^59^ and PHA-L as a β1,6-branched *N-*glycan binder.^59,60^ Our analysis is completely consistent with these annotations.

PHA-E from two sources (EY, Vector) show similar binding patterns, and machine learning analysis yields the expected motif of bisecting GlcNAc (Figure 4). In addition our analysis shows that core fucosylation and terminal α2,3-sialic acid structures are well tolerated, but α2,6-sialic acid inhibits binding. The only bisected type 1 LacNAc ligand on the array, a biantennary *N-*glycan, is not recognized by PHA-E. However, a nearly identical structure containing type 2 LacNAc is among the best binders, arguing that this lectin may discriminate between type 1 and type 2 LacNAc motifs.

The machine learning analysis of PHA-L from EY and Vector shows that, as expected, both bind preferentially to β1,6-branched *N-*glycans (Figure 4). In addition, our analysis found that binding of β1,6-triantennary structures is inhibited by α2,6- but not α2,3-sialic acid. Bisecting GlcNAc and core fucose are tolerated. Although β1,6-branched glycans containing type 2 LacNAc structures are well recognized, there are insufficient data to identify type 1 LacNAc structures as ligands.

#### *Robinia pseudoacacia* (RPA)

*Robinia pseudoacacia*, or black locust, has two lectins that have been isolated from its seeds.^61−63^ The commercially available preparation (E.Y. Laboratories) is a purified mixture of the seed proteins and is annotated as having complex specificity that is not inhibited by simple sugars. Machine learning shows that the principal binding determinant for RPA is multiantennary *N-*glycans containing a bisecting GlcNAc. Tetra-antennary are preferred over triantennary structures, and no binding is observed to biantennary structures. Core fucosylation enhances binding. Unlike other lectins in this group, binding is inhibited by α2,3- but not α2,6-sialic acid residues.

#### *Tulipa* Lectin (TL)

The primary lectin isolated from tulip bulbs (*Tulip sp.*, TL) has not been well characterized. Initial experiments using agglutination assays with monosaccharides and glycoprotein-based inhibition studies showed complex sugar specificity.^64^ Machine learning analysis indicates that the principal binders for TL are biantennary *N-*glycans. Binding affinity is strongly enhanced by core fucosylation. This lectin is fairly permissive in the composition of extensions and termini, including sialic acid substituents, fucosylation, GlcNAc, and LacNAc. However, bisecting GlcNAc has a negative impact on binding affinity. TL shows lower affinity binding to triantennary *N*-glycans but prefers β1,4- over β1,6-branched structures.

### Core *O*-Glycan Binding Lectins

The biosynthesis of canonical *O*-glycans begins with the transfer of a single sugar, *N-*acetylgalactosamine (GalNAc, Figure S1), onto serine or threonine. This epitope, known as the Tn antigen, can then be elaborated upon by a host of glycosyltransferases to make a variety of core structures. *O*-glycosylation is perhaps best studied on mucins, glycoproteins with clustered *O*-glycan sites that contribute to everything from lung function to cancer progression.^65^ Lectins that recognize *O-*glycans include *Amaranthus caudatus* (ACA, ACL), peanut agglutinin (PNA), *Artocarpus integrifolia* (AIA, Jacalin), *Codium fragile* (CF), *Maclura pomifera* (MPA, MPL), *Helix pomatia* agglutinin (HPA), and *Helix aspersa* agglutinin (HAA) (Figure 5). *Maackia amuerensis-II* (MAL-II), which binds sialic acid on *O-*glycans, is covered in Figure 7. In addition, the dual-specific lectin ABA binds select *O*-glycans (Figure 4). A detailed analysis of these lectins is given below.

#### *Amaranthus caudatus* (ACA, ACL)

The lectin from *Amaranthus caudatus* (ACL) is widely considered to bind T-antigen (Galβ1,3GalNAc-Ser/Thr).^66^ In contrast, our machine learning analysis shows that the best binders for ACL are core 1 and core 2 *O*-glycans, both of which contain the Galβ1,3GalNAc motif (Figure 5). Binding is enhanced by sulfation or sialylation at the 3 position of Gal and/or at the 6 position of the core GalNAc. Binding also tolerates other substitution on Gal, including fucose. Our analysis also revealed that a strong linker dependency for ACL recognition with no binding is observed when threonine (Sp14) is the linker. In contrast, the unbranched propyl amino-linker Sp8 (−CH_2_CH_2_CH_2_NH_2_) is strongly preferred. This suggests that ACL binds serine rather than threonine-linked *O-*glycans; however, it is not conclusive as serine is not used as a linker on this array. ACL also shows lower binding affinity to repeated Lewis structures (poly Lewis-x, *n* ≥ 2) or combinations of Le^x^, Le^y^, and Le^a^ antigens.

#### *Artocarpus integrifolia* (AIA, Jacalin)

The lectin isolated from *Artocarpus integrifolia* (aka Jackfruit: AIA, Jacalin)^67^ is widely considered a T-antigen binder. Our machine learning reveals this lectin predominantly binds a variety of 3-substituted GalNAcα epitopes (e.g., core 1 and core 3 *O*-glycans, Figure 5). A wide diversity of substituents are tolerated at the 3 position of GalNAc via either an α or β linkage, including GalNAc, GlcNAc, Gal, and longer oligosaccharides. However, binding is inhibited by any substitution at the 6 position of the core GalNAcα, precluding recognition of core 2, 4, 6 and 7 *O*-glycans. AIA from EY and Vector display identical binding patterns.

#### *Codium fragile* (Green Marine Algae, CF)

Isolated from the green marine algae, *Codium fragile* (CF) is reported to be a GalNAcα specific lectin.^68^ Machine learning analysis is consistent with these reports. The predominant binder is terminal GalNAcα, and it binds all epitopes containing this glycan including Tn and blood group A (Figure 5). CF also recognizes internal GalNAcα structures including core 1 and 3 *O*-glycans. However, unlike AIA, this lectin is insensitive to substitutions of the core GalNAc at the 6 position, as seen in core 2 and core 4 *O*-glycans, and the sialyl-Tn antigen. The lectin will also bind glycans containing terminal and internal GlcNAcα, which are not seen in mammals.

#### *Helix pomatia* Agglutinin (HPA) and *Helix aspersa* Agglutinin (HAA)

*Helix pomatia* agglutinin (HPA)^69^ and *Helix aspersa* agglutinin (HAA)^70^ are both commonly used as probes for GalNAcαSer/Thr (Tn antigen), an antigen with strong associations to cancer.^71^

The two lectins exhibit almost identical binding patterns. Machine learning shows a strong preference for GalNAcα-terminated oligosaccharides (Figure 5). Binding to GlcNAcα also emerges from our analysis, arguing that the stereochemistry at the 4-position of the terminal sugar is not essential to binding. HAA and HPA from two sources (EY, Sigma) show similar specificities across all four preparations.

#### *Maclura pomifera* (MPA, MPL)

The lectin from *Macluria pomifera* (osage orange: MPA)^72^ is considered a T-antigen binder. Our analysis found that MPA predominantly binds core 1 *O*-glycans (Figure 5) and can tolerate a wide variety substituents. Core 3 glycans, which contain GlcNAcβ1,3GalNAc, are also bound. However, binding is inhibited by substitution at the 6 position of the core GalNAcα (e.g., core 2).

#### Peanut Agglutinin (PNA)

Isolated from the peanut (*Arachis hypogaea*), PNA is commonly considered a T-antigen binder.^73^ Our analysis confirmed terminal Galβ1,3GalNAc as the preferred ligand for both preparations tested, although the lectin is insensitive to the anomeric linkage at GalNAc (Figure 5). This lectin also allows substitutions of the core GalNAc at the 6 position, as is seen in various core 2 *O*-glycans. However, binding is inhibited by any substitution on the Gal termini, arguing the requirement for unhindered access to terminal Galb1–3GalNAc for its binding.

### Lectins Binding Structures Common to N- and O-Glycans

*N*- and *O*-glycans carry a variety of epitopes beyond their core structures. These include terminal epitopes such as sialic acid and internal ones such as polyLacNAc. In this section, we discuss lectins that bind a range of epitopes found on *N-* and *O-*glycans. These include fucose (Figure 6), sialic acid and sulfate (Figure 7), terminal GlcNAc and chitin (Figure 8), terminal galactose and LacNAc (Figures 9 and 10), and terminal GalNAc (Figure 11).

#### Fucose Binding Lectins

Often considered a terminal modification, fucose is observed in diverse structural contexts within glycans and impacts an array of biological functions. Core fucosylation in mammals is exclusively α1,6-linked to the asparagine-linked GlcNAc in *N-*glycans and is observed on both hybrid and complex *N*-glycans (Figure S1).^32^ Core fucosylation impacts antibody-dependent cell-mediated cytotoxicity^74^ and cancer metastasis.^7^ α1,3- and α1,4-fucosylation are most commonly studied in the context of Lewis structures, including sialyl-Lewis^x^, which plays a role in inflammation.^75^ α1,2-fucose is a key component of blood group antigens. Lectins that predominantly recognize fucosylated glycans include *Aleuria aurantia* lectin (AAL), *Aspergillus oryzae* lectin (AOL), *Laburnum alpinum* lectin (LAA), *Lens culinaris* hemagglutinin (LcH, LcA), *Lotus tetragonolobus* lectin (LTL), *Pisum sativum* agglutinin (PSA), *Psophocarpus tetragonolobus* lectin-I (PTL/PTA I) and -II (PTL/PTA II), *Trichosanthes japonica* agglutinin II (TJA-II), and *Ulex europaeus* agglutinin-I (UEA-I) as shown in Figure 6. In addition, SNA-II (Figure 3), ACL (Figure 5), and UEA-II (Figure 8) can also recognize some fucosylated epitopes.

##### *Aleuria aurantia* Lectin (AAL) and *Aspergillus oryzae* Lectin (AOL)

The fungal lectins from *Aleuria aurantia* (AAL)^76^ and *Aspergillus oryzae* (AOL)^77^ are both known to bind fucose in many forms. Machine learning analysis shows that both lectins primarily bind α-linked fucose in many contexts and have very similar specificities (Figure 6). However, machine learning also revealed unexpected subtleties in their binding. Neither lectin recognized α1,2-fucose in the context of full epitopes of blood group A [GalNAcα1,3(Fucα1,2)Galβ1,3/4GlcNAc, BGA] or blood group B [Galα1,3(Fucα1,2)Galβ1,3/4GlcNAc, BGB]. There are also differences between the two lectins, with AAL showing a preference for Fucα1,2-terminated structures on type 2 over type 1 LacNAc, a finding not observed in AOL. In addition, only AOL was able to recognize type 3/4 blood group H antigens (Fucα1,2Galβ1,3GalNAc).

##### *Laburnum alpinum* Lectin (LAA)

*Laburnum alpinum* lectin (LAA) is known as a blood group H binder.^78^ Our analysis shows it prefers the type 2 blood group H epitope on *N*-linked glycans (Figure 6) and does not bind this epitope when presented on an *O*-glycan core. LAA is also reported to recognize *N*-acetylglucosamine/chitobiose.^79^ In keeping with this, we observed weaker binding to a complex mixture of other glycans. The majority of these structures contained a combination of β1,3-linkages and N-acetyl groups (e.g., GlcNAc β1,3Gal (in polyLacNAc), type 1 LacNAc, etc.).

##### *Lens culinaris* Hemagglutinin (LcH, LcA)

*Lens culinaris* hemagglutinin (LcH, LcA)^80^ is known to bind core fucose. Machine learning confirms this is the major binding determinant (Figure 6). Unexpectedly, we found that the presence of additional fucosylation in the glycan (e.g, α1,2-, α1,3-, α1,4-Fuc) or bisecting GlcNAc inhibits binding. The lectin tolerates a wide variety of terminal epitopes and branched structures. This is in contrast to previous work using frontal affinity chromatography that shows no binding for core fucosylated triantennary glycans.^81^ However, in that work, only structures branched at the β1,4- but not the β1,6-position were examined.^81^ The single β1,4-triantennary core fucosylated eptiope on CFGv5 does not bind LcH; however multiple examples of β1,6-triantennary core fucosylated epitopes are bound. Thus, it is likely that these lectins discriminate between β1,4- and β1,6-branching, although this cannot be definitively determined due to the lack of sufficient representative structures on this array. LcH also binds mannose structures containing terminal Manα1,2, although these are weak binders, in line with previous reports.^81^

##### *Lotus tetragonolobus* Lectin (LTL)

*Lotus tetragonolobus* lectin (LTL, Lotus) was originally annotated as a fucose-binding anti-H(O) lectin;^82,83^ however, more recent analysis has identified Lewis^x^ (Le^x^) as its main recognition motif.^84^ No rule was identified by machine learning; however α1,3-fucose was significantly enriched in LTL binding glycans, which included Le^x^ and Lewis^y^ (Le^y^, Figure 6, Table S4). Many other α1,3-fucosylated GlcNAc-containing glycans were not bound however, and the rules governing binding of this subset were not clear. No binding was observed to glycans bearing only α1,2-fucosylated glycans (e.g., blood group H).

##### *Pisum sativum* Agglutinin (PSA, PSL)

*Pisum sativum* agglutinin (PSA, PSL) was originally reported as a mannose binding lectin,^85,86^ although it is currently thought to bind core fucose. Its binding is closely related to LcH, and our analysis confirms core fucose as the major binding determinant (Figure 6). Although tolerant of a variety of structures, we found that the presence of bisecting GlcNAc inhibits binding. Mannose binding was not observed in our analysis but has been widely reported for this lectin.^87^

##### *Psophocarpus tetragonolobus* Lectin-I (PTL-I/PTA-I) and -II (PTL-II/PTA-II)

*Psophocarpus tetragonolobus* lectin *-*I (PTL-I) and -II (PTL-II) have distinct carbohydrate binding specificities.^88^ PTL-I is reported to be an GalNAcα-specific lectin.^89^ Rather than pan-GalNAcα, we found blood group A trisaccharides [GalNAca1,3(Fuca1,2)Gal] as the predominant binding motif for this lectin (Figure 6). The αGalNAc, is not strictly required for binding, as this lectin also recognizes blood group B, which has a terminal αGal residue. Binding is somewhat inhibited by additional α1,3-fucosylation on internal GlcNAc residues.

PTL-II is reported to recognize blood group H.^90^ In keeping with this, machine learning shows that PTL-II binds type 2 blood group H epitopes (Fuc α1,2Galβ1,4GlcNAc, Figure 6).

##### *Trichosanthes japonica* Agglutinin II (TJA–II)

*Trichosanthes japonica* yields two distinct lectins, *Trichosanthes japonica*-I (TJA-I), a sialic acid binder covered in Figure 7, and *Trichosanthes japonica*-II (TJA-II), which is annotated as a blood group H binder.^91^ Our analysis identified type 3/4 blood group H (Fucα1,2Galβ1,3GalNAc) as its predominant binding motif (Figure 6). Among the four types of blood group H antigens, H type 3/4 has the highest binding affinity, while types 1 and 2 are weaker binders, with a preference order: type 2 H ≫ type 1 H.

##### *Ulex europaeus* Agglutinin-I (UEA-I)

The gorse plant, *Ulex europaeus*, has two major lectins, *Ulex europaeus* agglutinin-I (UEA-I) and -II (UEA-II).^92−94^ UEA-I, annotated below, is a fucose lectin (Figure 6), whereas UEA-II is a chitin-binder (Figure 8).

UEA-I is well-known to recognize Fucα1–2Gal,^95^ and our analysis shows type 2 blood group H (Fucα1,2Galβ1,4GlcNAc) as the predominant binding epitope (Figure 6). No binding is observed to type 1 H epitopes (Fucα1,2Galβ1,3GlcNAc), indicating that the nature of the Gal linkage is important. Epitopes containing Glc (e.g., Fucα1,2Galβ1,4Glc) or subsituted on the GlcNAc (e.g., Le^y^) are tolerated. UEA-I was also found to tolerate sulfation on the 6-position of the terminal Gal.

#### Sialic Acid and Sulfate Binding Lectins

Both sialic acid and sulfation bring a negative charge to glycans. Sialic acid (Sia), also known as *N*-acetylneuraminic acid (Neu5Ac), can be found in a variety of linkages (α2,3-, α2,6-, and α2,8-) and is a terminal structure on *N-* and *O-*glycans and glycolipids. Abundant in the brain, sialosides modulate many processes, including neuronal migration, inflammation, and viral pathogenesis.^32^ Sulfation is often found in glycosaminoglycans, such as heparin, but is also critical on *N-* and *O-*glycans and glycolipids and has important roles in immunology.^96^ The following lectins bind to sialylated or sulfated structures on the array: cholera toxin B (CTB), *Maackia amurensis-I* (MAL-I, MAM, MAL), *Maackia amurensis-II* (MAL-II, MAH), *Polyporus squamosus* (PSL), *Trichosanthes japonica-I* (TJA-I), and *Sambucus nigra-I* (SNA-I; Figure 7). The specificity of these lectins is discussed in detail below.

##### Cholera Toxin B Subunit (CTB)

The B-subunit of cholera toxin (CTB) is commonly used to stain for the ganglioside GM1 [Galβ1,3GalNAcβ1,4(NeuAcα2,3)Galβ1,4Glc].^97,98^ Our analysis found only two glycan binders, GM1 and fucosylated GM1, in keeping with the known specificity of CTB (Figure 7).

##### *Maackia amurensis*-I (MAL-I, MAM, MAL) and -II (MAL-II, MAH)

Lectins isolated from the seeds of *Maackia amurensis* are commonly used to probe for α2,3 sialic acids.^99^ Two lectins have been identified from *Maackia*, MAL-I (MAM, MAL, often referred to as MAA) and MAL-II (MAH). Although these lectins have similar amino acid sequences (86.2% identity),^100^ they have distinct binding specificities.^99^ Machine learning analysis of MAL-I from three sources (E.Y. Laboratories, Vector Laboratories, and Seikagaku) shows that this lectin preferentially binds to terminal 3-O sulfated Gal on LacNAc (Figure 7). Although this has been observed previously,^13,99^ it is contrary to the common usage of this lectin as a sialic acid binder. Our analysis further identified fucosylation at the 3-position of GlcNAc (e.g., as in 3′*O*-sulfo Le^x^) as an inhibitory motif. Binding is enhanced by *O*-sulfation at the 6 position of GlcNAc. Although 3′*O*-sulfation is the strongest binding motif, only ∼30% of all binders are covered by this rule. In two of the three preparations of this lectin (Vector, Seikagaku), terminal α2,3-sialic acid on type 2 LacNAc is observed as a second determinant. Binding is again inhibited by fucosylation on GlcNAc (i.e., MAA-I does not bind sialyl Le^x^ or sialyl Le^a^). A variety of sialic acid variants were tolerated, including 5-*N*-glycolyl neuraminic acid (Neu5Gc), 9O,5N-diacetylated neuraminic acid (Neu5,9Ac) and KDN.

In contrast to MAL-I, the predominant binding determinant for MAL-II was defined by our analysis as α2,3-sialylated Galb1–3GalNAc in *O*-glycans (Figure 7). Various substitutions at the 6-position of GalNAc are tolerated, including sialylation, sulfation, and GlcNAc. This is in keeping with previous analysis.^99^ Similar to MAL-I, 3′O-sulfated Gal epitopes were observed to be an additional binding motif; however disulfation of galactose and/or fucosylation of the underlying residue are not tolerated.

##### *Polyporus squamosus* Lectin (PSL; Note, This Abbreviation Is Also Used by *Pisium sativum* Lectin)

The main lectin from *Polyporus squamosus* is known to bind α2,6-sialylated LacNAc.^101,102^ Machine learning confirms this as the principal binding motif of PSL, and all glycans on the array containing this motif, regardless of context, are recognized (Figure 7). This includes the single example of α2,6-sialylated type I LacNAc on the array. PSL tolerates Neu5Gc but does not bind to Neu5,9Ac.

##### *Sambucus nigra* Agglutinin (SNA, SNA-I)

One of several lectins isolated from elderberry bark (*Sambucus nigra*) SNA-I, also simply called SNA, is the most commonly used probe for α2,6-sialic acid.^103^ SNA was thought to require a disaccharide of the structure Neu5Acα2,6Gal/GalNAc. Machine learning reveals α2,6-sialylated LacNAc as the predominant binding determinant (Figure 7). As with PSL, the single case of type I α2,6-sialylated LacNAc is bound. No binding is observed to sialyl Tn antigen, in contravention of earlier literature.^103^ A wide variety of α2,6-sialic acids is tolerated (e.g., Neu5Gc, KDN), in keeping with previous findings.^104^

##### *Trichosanthes japonica*-I (TJA-I)

Isolated from *Trichosanthes japonica*, TJA-I is known to bind to Neu5Acα2,6Galβ1,4GlcNAc.^105^ Machine learning confirms this specificity and identifies the predominant binding motif as α2,6-sialylated LacNAc (Figure 7). The strongest binders present this motif on multiple antennae. TJA-I tolerates Neu5Gc and KDN. Weak binding is observed to the α2,6-sialylated type I LacNAc glycan.

#### Terminal GlcNAc and Chitin Binding Lectins

Terminal GlcNAc residues are common capping groups and are seen in a wide variety of glycan structures including chitin, a polymer of GlcNAcβ1,4GlcNAc. Several lectins predominantly recognize GlcNAc termini including *Griffonia simplicifolia-II* (GS-II), *Phytolacca Americana* (PWA), *Ulex Europaeus-II* (UEA-II), and wheat germ agglutinin (WGA; Figure 8). Other lectins, including several GalNAcaα binders (CF, HAA, HPA, Figure 5), UDA (Figure 4), and STA (Figure 9) also show binding to GlcNAc.

##### *Griffonia simplicifolia*-II (GS-II)

*Griffonia simplicifolia*-II (GS-II) is identified as a terminal GlcNAc binder.^106,107^ We confirmed terminal GlcNAcβ as the principal binding determinant (Figure 8). Our analysis shows the lectin prefers GlcNAc capped LacNAc in multiantennary *N-*glycans, or on polyLacNAc. Chitin oligomers [(GlcNAcβ1,4)n, *n* ≥ 2] and terminal GlcNAcα are also recognized. Two different preparations of GS-II (EY, Vector) have almost identical binding patterns.

##### *Phytolacca americana* (PWA)

The pokeweed plant (*Phytolacca americana*) has at least six lectins, all of which are annotated as chitin binders.^2^ Commercially available preparations of this lectin are a mixture of at least five of these proteins (E.Y. Laboratories). Machine learning analysis identifies chitin oligomers (GlcNAcβ1,4)_*n*_ (*n* ≥ 4) as the predominant binding motif for these lectins (Figure 8).

##### *Ulex europaeus* Agglutinin-II (UEA-II)

Unlike the fucose binding UEA-I, UEA-II is annotated as a β1,4-linked GlcNAc (i.e., chitin) binder.^108^ In contrast, our analysis identified terminal GlcNAcβ1,3Galβ as the predominant binding motif (Figure 8). All glycans containing this motif were bound, and no binding was observed to terminal chitobiose motifs. Binding to oligosaccharides containing GlcNAcβ1,3GalNAc and minor binding to H-antigen motifs were also observed.

##### Wheat Germ Agglutinin (WGA)

WGA, a lectin derived from wheat germ (*Triticum aestivum* or *Triticum vulgare*), is one of the most widely studied and commonly used lectins.^109^ Although WGA is often annotated as a GlcNAc binding lectin, it is thought to have a “broad” specificity, binding sialic acids and a mixture of GlcNAc containing glycans.^110^ We tested preparations from four vendors (EY, Vector, Seikagaku, Sigma) on CFGv5. As expected, machine learning indicates that the principal recognition motif for WGA is terminal GlcNAcβ (Figure 8). Presentation of this epitope on long chain polyLacNAc, multiantennary N-glycans, or longer chitin oligomers enhanced binding. In keeping with the literature, a wide variety of other terminal *N*-acetyl-containing glycans were also recognized, including terminal GlcNAcα-, GalNAcα-, GalNAcβ-, MurNAcβ-, and, in some preparations, Neu5Ac.

#### Terminal Gal and LacNAc Binding Lectins

Terminal galactose is observed in a wide variety of contexts, from the immunogenic α-Gal epitope to the ubiquitous Galβ structures of type 1 and type 2 LacNAc.^32^ The following lectins predominantly bind terminal galactose or LacNAc epitopes: *Bauhinia purpurea* lectin (BPA, BPL), *Erythrina cristagalli* agglutinin (ECA, ECL), *Griffonia simplicifolia*-I (GS-I), *Lycopersicon esculentum* agglutinin (LEA), *Marasmius oreades* agglutinin (MOA), *Pseudomonas aeruginosa*-IL (PA-IL), *Ricinus communis* agglutinin (RCA-I, RCA_120_), *Sophora japonica* agglutinin (SJA), and *Solanum tuberosum* lectin (STA, STL; Figure 9). The lectin from *Datura stramonium* (DSA) is also a LacNAc binder and is discussed earlier in this work as it is *N*-glycan specific (Figure 4). Additionally, UDA (Figure 3) and WFA (Figure 10) can also bind these epitopes. A detailed description of lectin binding specificities is discussed below.

##### *Bauhinia purpurea* Agglutinin (BPA, BPL)

*Bauhinia purpurea* agglutinin or lectin (BPA, BPL) was originally annotated as a GalNAc specific lectin,^111^ although more recent analysis has identified it as a T-antigen binder.^112^ In keeping with the more recent work, our analysis identified the principal binding determinant as terminal β-Gal, with a preference for β1,3 over β1,4 linkages (Figure 9). The underlying residue can be GlcNAc (as in type 1 LacNAc) or GalNAc (as in the T-antigen). This is one of the few lectins that shows a preference in binding for type 1 LacNAc. BPL tolerates substitution on the internal GlcNAc of LacNAc, including fucose. In addition, BPL recognizes terminal β-GalNAc attached to either Gal or in type 1 or type 2 LacdiNAc motifs, tolerating fucosylation on the internal GlcNAc. However, binding is inhibited by α2,3-sialylation on proximal Gal residues resulting in no recognition of GalNAcβ-terminated glycosphingolipids, including GM2 (GalNAcβ1,4(Neu5Ac α2,3)Galβ1,4Glc), GD2, GT2, and related structures.

##### *Erythrina cristagalli* Agglutinin (ECA, ECL)

*Erythrina cristagalli* agglutinin (ECA) was first identified as a Gal/GalNAc binding lectin.^113^ It is reported that ECA bound exclusively to various terminal LacNAc structures, polyLacNAc, and branched *O*-glycans.^114^ Our analysis lines up well with recent annotation of this lectin using multiple sources,^17^ identifying terminal type 2 LacNAc as the predominant binding epitope with enhanced binding observed with multivalent presentations (Figure 9). Also in keeping with this work, ECA was found to recognize unsubstituted terminal type 2 LacdiNAc (GalNAcβ1,4GlcNAc) and weak binding to epitopes containing terminal Fucα1–2Galβ1,4GlcNAc is seen.

##### *Griffonia simplicifolia*-I (GS-I)

GS-I, from *Griffonia simplicifolia*, is a mixture of five isolectins and is specific for α-galactosyl groups (Gal and GalNAc).^115,116^ In concordance with the known specificity, we identified the principal binding motif for GS-I as terminal α-Gal (Figure 9). In contrast to previous annotations,^2^ machine learning reveals inhibition of GS-I binding by α1,2-fucosylation on proximal residues, such as that seen in blood group B. Glycans containing terminal GalNAcα, including the Tn antigen, also bound GS-I.

##### *Lycopersicon esculentum* (LEA, LEL)

*Lycopersicon esculentum* agglutinin (LEA, LEL) binds GlcNAc oligomers, polyLacNAc, and/or chitin.^2,117^ In keeping with this, analysis of LEA from two different commercial sources (EY, Vector) showed overlapping binding patterns and identified both chitin chitin oligomers and type 2 polyLacNAc as dominant binding motifs. Our analysis revealed LEA binding is permissive for substitutions on LacNAc, including sialic acids, 3′O- or 6′O-sulfation, α1,2-fucosylation, and terminal GlcNAc. Type II LacdiNAc was also bound. In contrast, type I LacNAc was only recognized if the epitope was terminal on a type II LacNAc core, arguing that this motif is not recognized by the lectin.

##### *Marasmius oreades* Agglutinin (MOA)

The agglutinin from the mushroom *Marasmius oreades* (MOA) binds the xenotransplantation antigen Galα1,3Gal and blood group B.^118^ Our analysis concurs with the literature and identifies Galα1,3Gal as the predominant binding motif (Figure 9). This motif lies within the B trisaccharide, which is preferentially bound by this lectin. We found that MOA is sensitive to internal structures, as fucosylation of GlcNAc residues, as in Galα1–3Galb1–4(Fucα1–3)GlcNAc, inhibits binding. The closely related structure Galα1,3GalNAc is also recognized by MOA, in line with previous work.^119^

##### *Pseudomonas aeruginosa*-IL (PA-IL, LecA)

The bacterial lectin PA-IL, also known as LecA, from *Pseudomonas aeruginosa* exhibits affinity for α-galactosylated glycans.^120,121^ In keeping with this, our analysis identified the predominant binding motif as terminal α-Gal (Figure 9). However, we found that binding is inhibited by the presence of α1,2-fucosylation on the proximal residue, resulting in greatly diminished binding to blood group B.

##### *Ricinus communis* Agglutinin (RCA-I, RCA_120_)

Castor beans (*Ricinus communis*) contain two similar, but distinct, lectins: the potent cytotoxin ricin and the substantially less toxic RCA-I (RCA_120_).^122,123^ RCA-I from two commercial sources (EY, Vector) showed similar binding patterns. In keeping with a recent multidata source analysis,^17^ we identified terminal type 2 LacNAc as the main binding determinant and found that RCA-I tolerates substitution at the 6 position of the terminal galactose but not at the 3 position.

##### *Sophora japonica* Agglutinin (SJA)

*Sophora japonica* agglutinin (SJA) is known to bind both GalNAc and Gal (GalNAc > Gal), with an affinity for blood group B antigen.^124,125^ Machine learning identified terminal type 2 LacNAc on multiantennary branches or polyLacNAc chains containing >5 Gal residues as the predominant binding motif (Figure 9). This motif, however, only covered ∼1/3 of the binders. Glycans terminated with either blood group B or type 2 LacdiNAc were also strongly bound, indicating that all three motifs are recognized by this lectin.

##### *Solanum tuberosum* (STA, STL)

*Solanum tuberosum* agglutinin (STA, STL), isolated from potatoes, is reported to be a polyLacNAc and chitin binder.^2,126^ In keeping with this, we identified internal linear type 2 LacNAc as the major binding motif (Figure 9). Similar to LEA, STA tolerates a wide variety of termini, including sialic acid substituents. However, our analysis found that STA prefers linear glycans, and binding is diminished by branching (bi-, tri-, or tetra-antennary), indicating that binding requires free access to the linear chains. STA also binds chitin oligomers (*n* = 2–5) and type 2 LacdiNAc. Analysis of a second preparation of STA (EY) at a single concentration showed similar binding specificities.

#### Terminal GalNAc Binding Lectins

Exposed GalNAc residues are carried by a wide variety of oligosaccharides, including blood group A [GalNAcα1–3(Fucα1–2)Gal], LacdiNAc, the Forssman antigen (GalNAcα1–3GalNAc), and the Tn antigen (GalNAcαSer/Thr). The following lectins predominantly recognize GalNAc termini and are discussed below (Figure 10): *Cytisus scoparius* Lectin (CSA), *Dolichos biflorus* (DBA), soybean agglutinin (SBA), *Vicia villosa* lectin (VVL, VVA), and *Wisteria floribunda* agglutinin. Tn antigen binding lectins from *Codium fragile* (CF), *Helix aspersa* (HAA), and *Helix pomatia* (HPA) are discussed in Figure 5. In addition, several galactose specific lectins (BPA, ECA, GS-I, LEA, STA, Figure 9) also bind GalNAc terminal glycans.

##### *Cytisus scoparius* Lectin (CSA)

The *Cytisus scoparius* agglutinin CSA has been identified as a GalNAc specific lectin.^127^ In keeping with this, our analysis identified the predominant binder as terminal β-GalNAc (Figure 10). We found that CSA shows a preference for LacdiNAc epitopes, and binding to terminal β-GalNAc containing glycans is inhibited by the presence of sialylation or fucosylation at the 3 position of proximal residues. For example, glycosphingolipids such as GM2 are not recognized. A limited subset of simple mono- and disaccharide terminal α-GalNAc epitopes are also bound by this lectin.

##### *Dolichos biflorus* Agglutinin (DBA)

*Dolichos biflorus* agglutinin (DBA) is used as a probe for terminal α-GalNAc residues and is used to bind blood group A.^128^ In contrast to this, our machine learning analysis unequivocally identified the Forssman antigen as the best binding motif for all preparations of the lectin (Figure 10). This is in line with a previous report that identified this antigen as a far stronger binding epitope than blood group A.^129^ DBA also weakly bound to GM2 and related structures. Despite the literature, no significant binding to blood group A was observed except at the highest concentration of lectin tested, where weak binding could be seen, perhaps due to the presence of a far stronger binding motif.

##### Soybean (*Glycine max*) Agglutinin (SBA)

The lectin from *Glycine max* seeds, aka soybeans, is known as a GalNAc binder.^130,131^ Our analysis of two different preparations of SBA identify terminal β-GalNAc and the α-GalNAc terminated Forssman antigen as the predominant binding motifs (Figure 10). Similar to CSA, the lectin prefers β-GalNAc in terminal LacdiNAc glycans and is sensitive to glycosylation at the 3 position of the proximal residue. Weaker binding to α-GalNAc epitopes beyond the Forssman antigen is also observed, although fucosylation of the proximal residue, (e.g., as in blood group A) diminishes binding.

##### *Vicia villosa* Lectin (VVL, VVA)

The seeds of the hairy vetch plant, *Vicia villosa*, contain several lectins with distinct glycan binding specificities.^2^ The lectin commonly annotated as VVL (or VVA) is a GalNAc binder.^132,133^ The identified binding motifs for this lectin from two preparations, terminal β-*GalNAc* and *LacdiNAc*, are almost identical to those of CSA (Figure 10). Similar to CSA and SBA, binding is inhibited by fucosylation or sialylation of the proximal 3 position of β-GalNAc terminated glycans. Binding to a subset of simple mono- and disaccharide terminal α-GalNAc epitopes (e.g., Tn), but not the more complex blood group A, is also observed, concordant with literature reports.^133^

##### *Wisteria floribunda* Agglutinin (WFA, WFL)

The lectin from *Wisteria floribunda* (WFA, WFL) has been reported to recognize terminal *GalNAc* structures with high affinity, particularly those bearing LacdiNAc.^134,135^ In line with this, our analysis identified the principal binding motif as terminal β-*GalNAc* (Figure 10). Unlike other lectins in this group (CSA, SBA, VVL), we found that WFA is tolerant of substitution on the proximal residue and can bind β-*GalNAc* terminated glycosphingolipid structures*.* This lectin also recognizes simple terminal α-GalNAc epitopes. WFA shows significant binding to multiantennary glycans bearing terminal LacNAc epitopes (both type I and II), indicating that although terminal GalNAc is preferred, it is not absolutely required for binding.

## Conclusions

Lectins are a major tool for glycan analysis, finding use in flow cytometry, ELLA assays, lectin blots, histology, and lectin microarrays. Despite their ubiquitous presence in glycosylation research, proper annotation of their specificities is still limited. Herein, we have used a mixed machine learning and manual annotation approach to provide detailed annotation for 57 unique lectins using glycan microarray data.

In general, binding specificities obtained through glycan microarray analysis follow what is observed on cells, as demonstrated in recent work using cell-based arrays.^136,137^ In addition, structure-based modeling of lectin active sites is able to rationalize the majority of observed interactions.^15^ Limitations in our annotations are derived from the limitations of the array. Although the CFGv5 had a large number of glycans, it is missing glycans with nonuniform antennae, hybrid N-glycans, and multivalent presentations and is limited in its representations of some structures. Both the presence and presentation of ligands, including linkers, can have significant impacts on binding, especially at higher degrees of refinement.^13,14,138^ An emerging method to create more detailed annotations is the combining of data sets from multiple sources.^17^ As our analytical resolution of glycan binders improves, further insights will be gained into even finer aspects of binding.

The lectins annotated herein cover the majority of those used in the literature. In general, our analysis found good concordance between preparations of the same lectin, regardless of source, with some lot to lot variation, which are most likely due to differences in the natural products. This points to the importance of cross-validating lectin results from naturally isolated lectins, for example through the use of an array with multiple probes. It also showcases the need for more recombinant lectins and antibodies.^139−141^

Our analysis brings new insights into lectin specificities, finding both known binding motifs and previously unknown requirements for lectin binding. For example, MAA-I, commonly used to detect α2,3-sialic acid, was confirmed to preferentially bind 3′-O sulfation as previously reported. Machine learning identified that this lectin is inhibited by fucosylation of the proximal GlcNAc, as in sialyl or sulfo-Lewis x, a new finding. The more detailed annotation provided by this work presents a guide to their use and sets the stage to garner additional insights from lectin binding. This includes more advanced annotation of motifs from lectin microarrays and other multilectin studies.

## Materials and Methods

### Lectins and Antibodies

Biotinylated lectins were purchased from the following companies: E.Y. Laboratories, Vector Laboratories, Seikagaku Corporation, and Sigma. Lectins that were not available in biotinylated form were biotinylated using the Pierce Biotinylation kit (Pierce) and standard methods. For a complete list of lot numbers and vendors for lectins, please see Supplemental Table S2.

### Glycan Microarray Analysis

All lectins were analyzed in the Consortium for Functional Glycomics Glycan microarray, version 5.0 as detailed in ref (13). Lectins that did not show binding for any glycan ≥4000 RFUs at the concentrations tested, or that had data for only a single concentration of a unique lectin with signals <4000 RFU, were removed from further analysis. The raw data are available as a collection on Synapse.org (DOI: 10.7303/syn26469702) and at http://www.functionalglycomics.org/static/index.shtml (search keyword Mahal to find all data sets).

### Data Analysis

We generated *Z*-score data for each glycan microarray, excluding those whose highest signal was <1000 RFU. For each GBP, we then generated a master *Z* score (*Z*_s_) using *Z*-score data from multiple concentrations via Stouffer’s method.^29^ In brief, the *Z* scores for a glycan across concentrations were summed and divided by the square root of the number of concentrations. Calculations were done using Microsoft Excel 2011. Only GBPs for which multiple concentrations were tested and had more than a single array with signals >1000 RFU were considered in our analysis. We used a threshold of *Z*_s_ = 1.645 (*p* ≤ 0.05) to establish significance. Glycans that failed to meet this threshold for all GBPs tested were removed from our analysis (see Table S1). For each lectin, glycans that met our *Z*_s_ threshold but did not vary across concentrations (σ^2^ < 10% of maximum variance across arrays) were set to average prior to generation of the hierarchical cluster and were flagged for careful consideration in our annotation. If these glycans gave signals of <10% of the maximum signal at the highest lectin concentration, they were considered nonspecific binders in our motif analysis. Data were then annotated by hand to identify binding motifs. A heatmap was generated using Cluster 3.0 using the Pearson correlation coefficient as the distance metric and average linkage analysis and visualized with Java Treeview.

### Data Processing

For each glycan, we subtracted the average binding *Z* score across all experiments to correct for unspecific background binding. We removed several glycans from downstream analysis as their binding pattern indicated unspecific binding (Table S1).

### Hierarchical Clustering to Determine Lectin Binding Specificity

Using the processed *Z*_s_ data, we generated hierarchically clustered heatmaps for all remaining glycans. For this, we removed several lectins that did not show a well-defined binding pattern (LPA_EY, NPA_EY, VRA_EY, BDA_EY, SJA_EY, GS-I_EY, HMA_EY, PWA_EY, TKA_EY, SVAM_Sigma, ACL_EY, LTL_EY). In addition, LcH_EY was clearly mislabeled as it was almost identical to AIA. Therefore, these lectins were removed from subsequent analysis. The heatmap was then generated using the Multiexperiment Viewer software (MeV_4_8, v.10.2). Samples were clustered heiarchically using the Pearson correlation as the distance metric and average linkage analysis.

### Extracting Glycan-Binding Rules for Lectins Using Machine Learning

We used the Python package SkopeRules to identify logical, interpretable rules for lectin binding specificity. This procedure extracts rules from ensembles of tree-based machine learning models^142^ that explain the glycan binding behavior of a given lectin using glycan features as input variables. The underlying machine learning models were trained on 80% of the processed data to predict whether a given glycan was bound (*Z* score ≥1.645) in the remaining 20% of the data. Our ensembles consisted of 50 estimator models with a depth of one. As input features for these models, we used both 68 hand-annotated glycan features (refer to Supporting Information Table) as well as the count of all observed mono- and disaccharide motifs and linkages. For each rule, we used the default thresholds recommended by SkopeRules of 0.3 for precision and 0.1 for recall to determine if a rule is valid. In this context, precision refers to the fraction of glycans fulfilling a given rule that bind to the lectin of interest, while recall indicates how many of all bound glycans for that lectin fulfill the rule. Additionally, we only considered rules that had been chosen by the algorithm in at least 15% of the estimators to increase robustness. If no rule that fulfilled these requirements could be identified at a depth of one, we searched for rules among estimators with a maximum depth of two. If two rules were identified that both satisfied our quality criteria, we indicated them as alternative rules on the same level (e.g., rule 1a and 1b). After identifying the first rule, we selected all glycans fulfilling that rule and searched for another rule that improved the prediction of the glycan binding behavior. We repeated this procedure until we either could not identify another rule with our minimal requirements, reached a precision of 1.0, or reached five levels of rules.

## Supporting Information Available

Overview of N- and O- glycans (PDF)
Table of annotated motifs (XLSX)
Additional vendor and lectin lot information (XLS)
Machine-learning rules (XLSX)
Motif-associated *p* values (XLSX)
*z*-score analysis of lectins (XLSX)
