# Supplementary material for: A Useful Guide to Lectin Binding: Machine-Learning Directed Annotation of 57 Unique Lectin Specificities
Source: ACS Chem Biol. 2022 Jan 27;17(11):2993–3012. doi: 10.1021/acschembio.1c00689 (PMC9679999; doi:10.1021/acschembio.1c00689)
Supplement: Supplementary file 1 — cb1c00689_si_001.pdf [file cb1c00689_si_001.pdf]

SUPPORTING INFORMATION

A Useful Guide to Lectin Binding: Machine-Learning Directed Annotation of 57 Unique Lectin Specificities

Daniel Bojar [1¥], Lawrence Meche[2¥], Guanmin Meng[3¥], William Eng[2], David F. Smith[4], and Richard D. Cummings[5], Lara K. Mahal[2,3*]

1. Department of Chemistry and Molecular Biology, University of Gothenburg, Gothenburg, Sweden. Wallenberg Centre for Molecular and Translational Medicine, Gothenburg, SWEDEN 405 30.

2. Biomedical Chemistry Institute, New York University Department of Chemistry, 100 Washington Square East, Room 1001, New York, NY, USA, 10003.

3. Department of Chemistry, University of Alberta, Edmonton, CANADA, T6G 2G2.

4. Department of Biochemistry, Glycomics Center, Emory University, School of Medicine, Atlanta, GA, USA, 30322.

5. Department of Surgery, Beth Israel Deaconess Medical Center, Harvard Medical School, Boston, MA, USA, 02115.

¥ Authors contributed equally

* To Whom Correspondence Should be Addressed. lkmahal@ualberta.ca

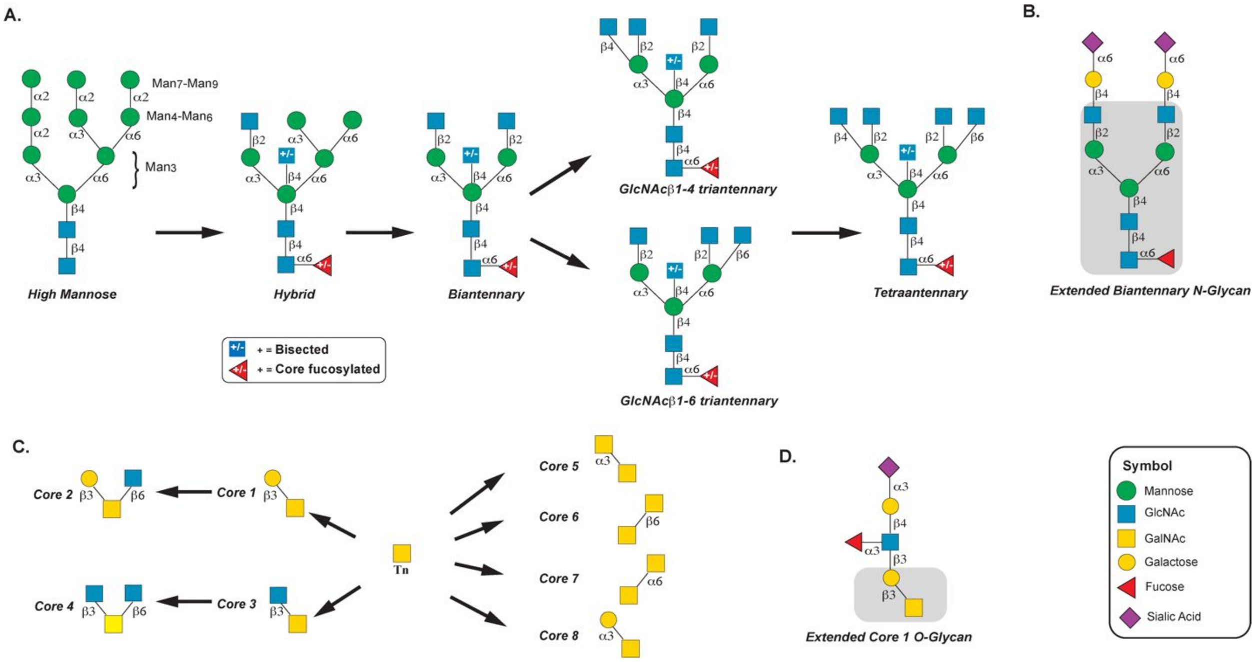

Figure S1. Overview of *N*- and *O*- glycans. A. *N*-glycosylation pathway. High mannose is trimmed Man_5 and then elaborated to hybrid, biantennary, triantennary and tetraantennary *N*-glycans. B. An example of a more complex biantennary *N*-glycan. The biantennary core is highlighted in grey. C. *O*-glycan cores. D. An example of a more complex core 1 O-glycan. The core is highlighted in grey.

Tables S1-5: Excel files.

Table S1: Annotated List of Glycans With Curated Motifs

Table S2: List of Lectins Analyzed

Table S3: Machine Learning Rules for Lectins

Table S4: Corrected p-values for Significant Motifs

Table S5: Zs Analysis of Lectins with Motifs Shown
